# Irisin improves adiposity and exercise tolerance in a rat model of postmenopausal obesity through enhancing adipo-myocyte thermogenesis

**DOI:** 10.1007/s13105-022-00915-3

**Published:** 2022-08-23

**Authors:** Rehab E. Abo El Gheit, Reham L. Younis, Mervat H. El-Saka, Marwa N. Emam, Nema A. Soliman, Rehab M. El-Sayed, Yasser Mostafa Hafez, Norhan Ahmed AbuoHashish, Doaa A. Radwan, Howayda E. khaled, Samar Kamel, Sawsan A. Zaitone, Ghada A. Badawi

**Affiliations:** 1 Department of Physiology, Faculty of Medicine, Tanta University, El Geesh Street, Tanta, Egypt; 2 Medical Biochemistry Department, Faculty of Medicine, Tanta University, Tanta, Egypt; 3 Department of Pharmacology & Toxicology, Faculty of Pharmacy, Sinai University, North Sinai, El-Arish, Egypt; 4 Internal Medicine Department, Faculty of Medicine, Tanta University, Tanta, Egypt; 5 Pharmacology Department, Faculty of Medicine, Tanta University, Tanta, Egypt; 6 Anatomy and Embryology Department, Faculty of Medicine, Tanta University, Tanta, Egypt; 7 Zoology Department, Faculty of Science, Suez Canal University, Ismailia, Egypt; 8 Physiology Department, Faculty of Veterinary Medicine, Suez Canal University, Ismailia, Egypt; 9 Department of Pharmacology & Toxicology, Faculty of Pharmacy, Suez Canal University, Ismailia, 41522 Egypt; 10 Department of Pharmacology & Toxicology, Faculty of Pharmacy, University of Tabuk, Tabuk, 71451 Saudi Arabia

**Keywords:** Irisin, Postmenopausal obesity, Mitochondrial uncoupling protein-1, Sarcolipin, Sarco-endoplasmic reticulum Ca^2+^-ATPase

## Abstract

The prevalence of obesity and its associated metabolic disorders, along with their healthcare costs, is rising exponentially. Irisin, an adipomyokine, may serve as a critical cross-organ messenger, linking skeletal muscle with adipose tissue and the liver to integrate the energy homeostasis under diet-induced obesity. We aimed to explore the putative role of irisin in the protection against obesity in a postmenopausal rat model by modulating energy expenditure (EE). Bilateral ovariectomy (OVX) was performed. After 3 weeks of recovery, the OVX rats were classified according to their dietary protocol into rats maintained on normal diets (ND) (OVX) or high-fat diet (HFD) groups. The HFD-fed animals were equally divided into OVX/HFD, or irisin-treated OVX/HFD groups. Sham rats, maintained on ND, were selected as the control group. We evaluated anthropometric, EE, and molecular biomarkers of browning and thermogenesis in inguinal white adipose tissue and skeletal muscle, and the activity of the proteins related to mitochondrial long chain fatty acid transport, oxidation, and glycolysis. HFD of OVX further deteriorated the disturbed glucose homeostasis, lipid profile, and the reduced irisin, thermogenic parameters in adipose tissue and skeletal muscle, and EE. Irisin treatment improved the lipid profile and insulin resistance. That was associated with reduced hepatic gluconeogenic enzyme activities and restored hepatic glycogen content. Irisin reduced ectopic lipid infiltration. Irisin augmented EE by activating non-shivering thermogenesis in muscle and adipose tissues and decreasing metabolic efficiency. Our experimental evidence suggests irisin’s use as a potential thermogenic agent, therapeutically targeting obesity in postmenopausal patients.

**Graphical abstract:**

Irisin modulates the non-shivering thermogenesis in skeletal muscle and adipose tissue in postmenopausal model

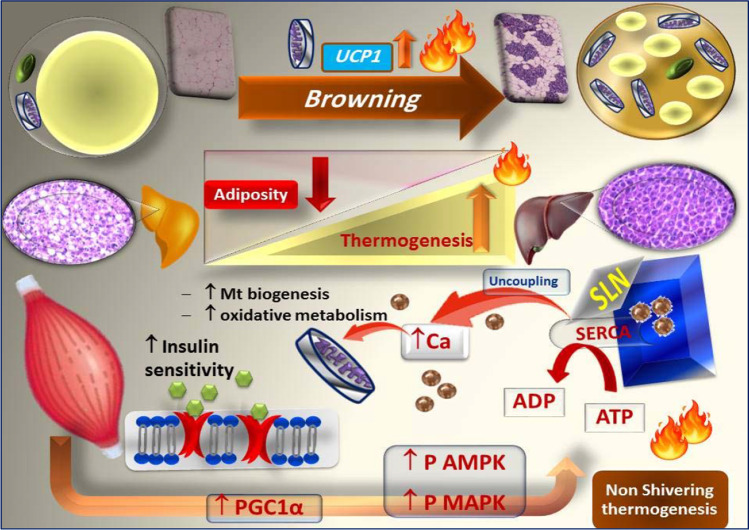

**Supplementary Information:**

The online version contains supplementary material available at 10.1007/s13105-022-00915-3.

## Introduction

Obesity is characterized by excessive fat buildup when total energy expenditure (EE) persistently exceeds total energy intake (EI) [1, 2]. Among several implications that are closely linked with obesity, menopause deserves special attention. The abrupt hormonal changes associated with the loss of ovarian function are responsible for a variety of metabolic and physical abnormalities that have a negative impact on women’s health and quality of life [3].

In contrast to white adipose tissue (WAT), which is concerned with energy storage, brown adipose tissue (BAT) is an important site of EE with a higher efficiency for energy dissipation via mitochondrial uncoupling protein-1 (UCP-1)-mediated thermogenesis. Enhanced BAT activity is linked with considerable lipid and glucose disposal [2], whereas dysregulation of BAT thermogenic ability significantly lowers EE and promotes fat development [1]. Adults have considerable levels of inducible BAT, as demonstrated by beiging. Beige cells have a unique molecular profile that comprises the production of a core set of brown fat gene markers that, when activated, result in enhanced uncoupled respiration [2].

Decreased BAT mass and activity have been observed in elderly and obese individuals [4]. As a result, encouraging non-shivering thermogenesis (NST) might be developed to counteract postmenopausal obesity, particularly at the ambient thermal scale.

Skeletal muscle is the largest organ in the body and the primary determinant of the basal metabolic rate (BMR), besides its ability to consume up to 80% of insulin-stimulated glucose uptake.

Indeed, skeletal muscle is more than a contractile machine. Muscle is the fundamental thermogenic organ, since its contraction is coupled with the production of heat that can be exploited by shivering, in addition to its crucial involvement in adaptive thermogenesis in response to diet overload. Although accumulating evidence suggests skeletal muscle capacities for NST, it remains poorly understood [5].

The sarcoplasmic reticulum (SR) calcium (Ca^2+^) cycling regulates the duration and amplitude of skeletal muscle contractions. It also serves as a signal to the mitochondria allowing it to synchronize its coupling of energy demand and energy supply. Failure to keep this delicate balance results in reduced force, which is perceived as fatigue. Furthermore, the disturbed energy balance might considerably lead to excess weight gain. SR (Ca^2+^) ATPase (SERCA) is one of the (Ca^2+^)-handling proteins that control SR Ca^2+^ uptake and release [5].

Sarcolipin (SLN) is a SERCA pump regulator that has recently been discovered as a key player in muscle metabolism and thermogenesis. SERCA pump is encoded by two genes isoform: SERCA1 (predominant in fast-twitch muscle) and SERCA2 (abundant in slow oxidative fibers) [5].

Myocytes release multiple myokines in response to the skeletal muscle contraction, which are involved in metabolic regulation within the muscle in an autocrine manner and in distant organs, such as the adipose tissue and liver, in an endocrine or paracrine fashion. Irisin is a newly discovered exercise-induced adipo-myokines that regulates energy homeostasis. Irisin is an extracellularly photolytic cleavage product shed into circulation from the parent polypeptide, fibronectin type III domain containing 5 (FNDC5).

Irisin is highly conserved among all mammalian species sequenced. Human and rat irisin are 100% similar, implying a highly conserved function mediated mostly by a cell surface receptor [6]. While several experimental studies have attempted to delineate the role of irisin in adults’ health, the significance of irisin during postmenopausal life, particularly when confounded by caloric overload, has often been underexplored. So, we aimed, in the current study, to uncover the potential role of irisin in postmenopausal obese rats and the possible underlying molecular mechanisms. Further, we highlighted the potential contribution of UCP1 and SLN/SERCA based NST in adipose and muscle tissues, respectively, in exploiting the EE.

## Research design and methods

### Experimental animals

Forty, 3-month-old female albino rats (weight: 200 ± 10 g on average) were purchased from the Faculty of Science, Tanta University. All rats were appropriately housed in pairs in a stainless steel cage in a room maintained at 50% relative humidity and 25 ± 2 °C with a 12/12-h light–dark cycle. From the start of our study till the end of the 3-week recovery post ovariectomy, all experimental animals were fed distilled water and commercialized pelletized chow ad libitum to stabilize their metabolic condition.

### Ethics standards

All experimental procedures were conducted in compliance with the institutional principles of Laboratory Animal Care, as well as the specific international guidelines, and were approved by the Research Advisory Ethical Committee of the Faculty of Medicine, Tanta University, Egypt (approval no. 33901/6/20).

### Induction of menopause

Bilateral surgical ovariectomy was performed under ketamine anesthesia (ketamine hydrochloride, 75 mg/kg body weight (BW), i.p.) [7].

During the sham procedure, rats were anesthetized and the abdominal wall was opened, similar to that used for the ovariectomized (OVX) animals. Then the ovaries were manipulated and exteriorized to create similar stress, but they were not removed. Immediately after surgery, all the rats received penicillin and ibuprofen 50 mg (0.1 ml/kg body weight) for 2 days, to minimize the rats’ postoperative infection and pain. Then the rats were allowed a 3-week recovery period, after which a cytological examination of vaginal smears from all OVX rats was carried out to ensure the surgically induced menopause. The uterine weight was measured immediately after decapitation to ensure the success of the ovariectomy [7, 8].

### Dietary regimen

At the end of the recovery period, obesity was established by following an 8-week dietary regimen [7]. According to the dietary protocol, the OVX rats were divided into two groups based on the dietary protocol: normal diet (ND) or high-fat diet (HFD) groups, respectively. Each group was maintained on its diet regimen till the end of the study.

In the ND fed groups [group I (control group) and group II (OVX-ND)], the animals were daily fed with a standard semisynthetic diet that was formulated to provide all the required nutrients, including minerals and vitamins, for normal growth in rats as recommended by the American Institute of Nutrition (AIN-93 M) dietary guidelines [9].The HFD-fed animals were equally divided into two weight-matched groups [group III (OVX/HFD) and group IV (irisin-treated OVX/HFD rats)]. ND is composed of 10% fat, 76% carbohydrate, and 14% protein, while HFD is composed of 60% fat, 26% carbohydrate, and 14% protein, based on percentage of total calories. The vitamin and mineral compositions of ND and HFD were identical [8].

The experimental diets were prepared weekly and stored at 4 °C. The supplement (Table 1) depicts the full composition of each diet and the related modifications to the energy levels to formulate HFD.

**Table 1:** Effect of irisin on anthropometric, obesity-related parameters, and exhaustive swimming exercise, in an obese postmenopausal rat model

Parameters	Group			
	Group I	Group II	Group III	Group IV
	Control	OVX	OVX/HFD	Irisin-treated OVX/HFD
Initial BW (g)	200 ± 12	202 ± 10	199 ± 13	203 ± 12
Final BW (g)	281.5 ± 10	322.7 ± 15^*^	439.9 ± 14^*#^	287.8 ± 18^#§^
Delta weight gain	81.5 ± 7.1	120.7 ± 9.4^*^	240.9 ± 11.4^*#^	84.8 ± 4.5^#§^
BW gain percent (BWG %)	40.8 ± 6.4	59.8 ± 4.8^*^	121.1 ± 9.2^*#^	41.8 ± 5.1^#§^
BMI (g/cm^2^)	0.53 ± 0.02	0.645 ± 0.03^*^	0.948 ± 0.04^*#^	0.576 ± 0.01^#§^
Food intake (g)/day/rat	12 ± 0.62	19 ± 0.83^*^	30 ± 0.71^*#^	28 ± 0.82^*#^
Calorie intake of consumed diet (kcal/day)	42.88 ± 3.4	102.676 ± 9.8^*^	162.12 ± 10.2^*#^	151.312 ± 10^*#^
Caloric efficiency (weight gain (g)/g of diet consumed)	0.0849 ± 0.005	0.0794 ± 0.002	0.1004 ± 0.02^*#^	0.0379 ± 0.001^*#§^
Total fat pad mass (g)	40.7 ± 6.4 (15%)	93.4 ± 7.9^*^ (29%)	182.4 ± 10.8^*#^ (42%)	37.2 ± 5.3^#§^ (13%)
LBM (g)	234.9 ± 10.5 (82.5%)	228.7 ± 9.7 (69%)	245.5 ± 13.9^*#^ (56%)	243.7 ± 6.7^*#^ (85%)
Exhaustive swimming exercise: time to exhaustion (min)	8.13 ± 1.3	5.673 ± 1.1^*^	3.22 ± 0.58^*#^	12.57 ± 2.16^*#§^

Values are expressed as mean ± SD of 10 rats in each group

^*^Significance vs. control group (*P* < 0.05)

^#^Significance vs. OVX group (*P* < 0.05)

^§^Significance vs. OVX/HFD group (*P* < 0.05)

*BMI* body mass index, *BW* body weight, *BWG* body weight gain, *LBM* lean (fat-free) body mass, *OVX* ovariectomized, *OVX/HFD* ovariectomized/high-fat diet fed group

**Fig. 1:**
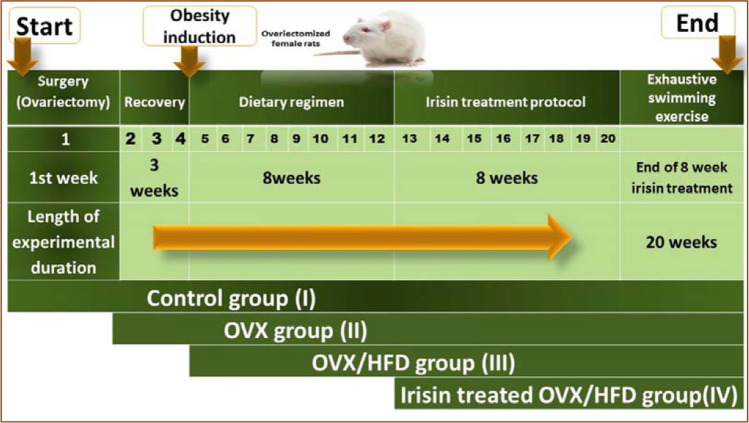
Schematic representation of the time line and the experimental design utilized in the present study. In the first week, bilateral surgical ovariectomy was performed, followed by a 3-week recovery period. At the start of the fifth week, obesity was established by an 8-week dietary regimen for ovariectomized (OVX) rats. OVX rats were classified—according to their dietary protocol, into normal diet (ND, group II (OVX)) or high-fat diet (HFD). Each group was maintained on its diet regimen till the end of the study. The HFD-fed animals were equally divided into two weight-matched groups [group III (OVX/HFD) and group IV (irisin-treated OVX/HFD)], starting from the 13th week and continued for 8 weeks later. At the end of the experiment, all rats were subjected to exhaustive swimming exercise. *N* = 10 rats/group

Grouping: 40 rats were divided equally into four groups (10 rats each) (Fig. 1).

Group I (sham-operated (control) group): non-OVX (control) rats that were fed ND throughout the experimental period.

Group II (OVX group): The rats underwent ovariectomy. After the 3-week recovery period, the OVX rats were kept on ND after surgery till the end of the experiment.

Group III (OVX-HFD group): The rats underwent ovariectomy. After the 3-week recovery period, the OVX rats were kept on HFD, starting from the 5th week till the end of the experiment.

Group IV (irisin-treated OVX-HFD group): The rats underwent ovariectomy. After the 3-week recovery period, the OVX rats were kept on HFD from the 5th week, and they were concomitantly treated with recombinant (r)-irisin (0.5 μg/g BW, daily; i.p.; Abnova, Taiwan, China), starting from the 13th week and continuing for 8 weeks later. The r-irisin dose was determined in the light of previous reports [10]. The other groups received the same volume of saline injections.

Detailed methodology, sampling, and processing, biochemical and molecular analysis: available in supplementary data.

### Statistical analysis

Results were expressed as means ± standard deviation (SD) of 10 rats per group, for each parameter (except the relative gene expression and western blot, which had 6 rats each). Statistical analysis was performed by one-way analysis of variance (ANOVA) followed by a post hoc test of Tukey’s test using GraphPad Prism 4.03 (GraphPad Software, San Diego, CA, USA). *P* values less than 0.05 were considered statistically significant.

## Results

### The effect of irisin on anthropometric measurements, caloric intake/efficiency, feeding behavior, and core body temperature in an obese postmenopausal rat model

The OVX group reported a 59.8% rise in BW, compared to 40.8% in the control group, whereas the OVX/HFD group experienced a 121.1% increase in BW. That was linked with increased body mass index (BMI) of 0.645 ± 0.03 and 0.948 ± 0.04 in the OVX and OVX/HFD groups, respectively, in contrast to a BMI of 0.53 ± 0.02 in the control group (Table 1).

**Fig. 2:**
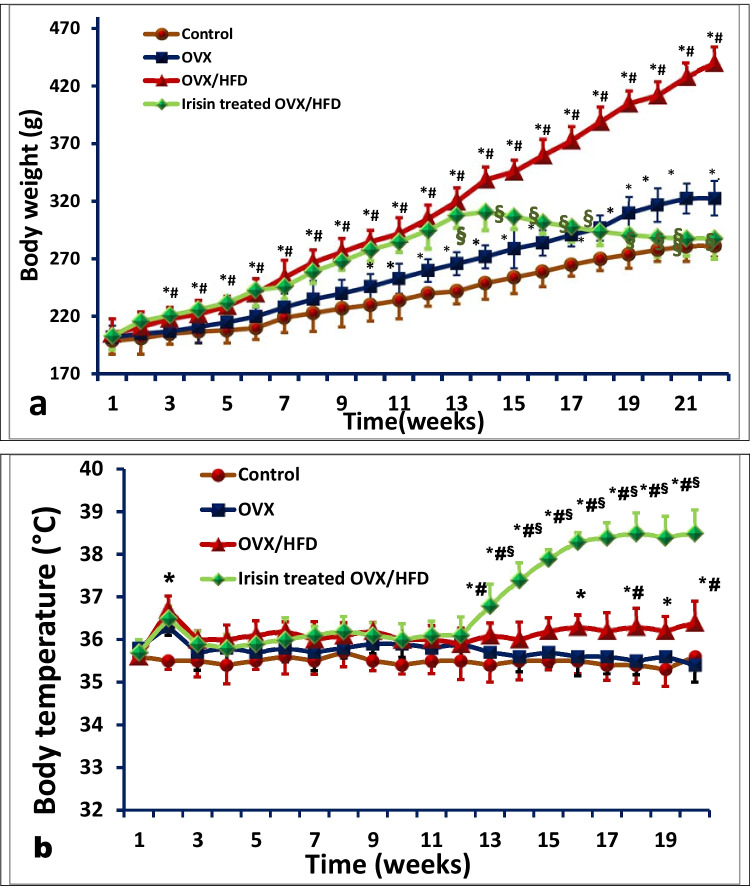
The effect of irisin on **a**) body weight gain and **b**) core body temperature, in an obese postmenopausal rat model. Body weight (a) and core body temperature (°C) (b) were measured weekly. Values are expressed as mean ± SD of 10 rats in each group. *Significance vs. control group (*P* < 0.05). ^#^Significance vs. OVX group (*P* < 0.05). ^§^Significance vs. OVX/HFD group (*P* < 0.05)

The increased BW in the OVX group was associated with an increase in the total fat pad mass to about 29% of BW compared to 69% lean body mass (LBM), while the OVX/HFD group displayed marked expansion in adipose tissue to approach 42% of BW with a concomitant reduction in LBM to 56% of BW (Table 1, Fig. 2). Irisin treatment resulted in a reduction in BMI to 0.576 ± 0.01, a level comparable to that of the control group (0.53 ± 0.02), with an obvious increase in LBM to 85% at the expense of 13% fat weight of BW compared to 15% and 82.5% fat weight and LBM in the control group (Table 1).

In comparison to the control and OVX groups, OVX/HFD significantly increased food intake and caloric efficiency. Despite a continued rise in total calorie intake, irisin therapy resulted in a significant decrease in caloric efficiency (Table 1).

In terms of core body temperature, the enhanced metabolic milieu and thermogenic parameters, as well as oxidative metabolism, in the irisin-treated OVX/HFD group were linked with a substantial rise (38.34 ± 0.45) compared to the OVX/HFD (36.46 ± 0.64), OVX (35.47 ± 0.46), and control group (35.9 ± 0.5) (Fig. 2).

### The effect of irisin on glucose homeostasis in an obese postmenopausal rat model

Obesity induction by HFD exacerbated the hyperglycemia and IR that developed with surgically induced menopause in group III. The homeostasis model assessment of insulin resistance (HOMA-IR) was substantially higher in groups II and III, respectively, at 2.7 ± 0.23 and 8.5 ± 1.31 compared to 1.5 ± 0.19 in the control group. This was linked to 1.4 and 1.7 increases in insulin levels in groups II and III, respectively, as well as hyperglycemia (Table 2).

**Table 2:** Effect of irisin on serum, muscle, and liver biochemical metabolic parameters in an obese postmenopausal rat model

Parameters	Group			
	Group I	Group II	Group III	Group IV
	Control	OVX	OVX/HFD	Irisin-treated OVX/HFD
Serum irisin (ng/ml)	3.110 ± 0.32	2.140 ± 0.18^*^	1.241 ± 0.09^*#^	3.050 ± 0.23^#§^
Glucose (mg/dl)	81.24 ± 3.09	109.0 ± 4.5^*^	194.3 ± 9.4^*#^	129.7 ± 4.1^*#§^
Fasting serum insulin (µIU/ml)	7.303 ± 0.91	10.55 ± 1.2^*^	17.85 ± 2.5^*#^	8.536 ± 1.02^#§^
HOMA-IR	1.5 ± 0.19	2.7 ± 0.23^*^	8.5 ± 1.31^*#^	2.9 ± 0.13^*§^
Serum TG (mg/dl)	62.09 ± 6.82	95.80 ± 7.13^*^	155.6 ± 9.16^*#^	88.90 ± 4.452^*§^
Serum TC (mg/dl)	88.90 ± 5.8	125.3 ± 9.3^*^	181.7 ± 7.0^*#^	100.0 ± 3.4^#§^
Serum FFA (mmol/L)	0.27 ± 0.3	0.53 ± 0.1^*^	1.07 ± 0.17^*#^	0.36 ± 0.04^#§^
GM glycogen (mg/g tissue)	9.5 ± 2.1	6.3 ± 1.7	4.9 ± 1.10^*^	12.91 ± 2.8^*#§^
Liver glycogen (mg/g tissue)	52.3 ± 6.4	38.4 ± 4.6^*^	28.9 ± 3.9^*#^	67.5 ± 9.05^*#§^
Liver TG (mg/g tissue)	26.20 ± 4.2	51.80 ± 5.4^*^	235.6 ± 10.2^*#^	44.30 ± 3.2^*§^

Values are expressed as mean ± SD of 10 rats in each group

^*^Significance vs. control group (*P* < 0.05)

^#^Significance vs. OVX group (*P* < 0.05)

^§^Significance vs. OVX/HFD group (*P* < 0.05)

*FFA* free fatty acids, *GM* gastrocnemius, *HOMA-IR* homeostasis model assessment of insulin resistance, *OVX* ovariectomized, *OVX/HFD* ovariectomized/high-fat diet fed group, *TC* total cholesterol, *TG* triglycerides

Irisin administration resulted in an obvious improvement in IR, with glucose, insulin, and HOMA-IR levels reduced to levels comparable to the control group (Table 2).

### The effect of irisin on muscle glycogen content in an obese postmenopausal rat model

Both OVX and OVX/HFD were associated with a depletion of glycogen stores in muscle (6.3 ± 1.7 and 4.9 ± 1.10) as compared to the control group (9.5 ± 2.1). Irisin therapy increased muscle glycogen content significantly (Table 2).

### The effect of irisin on liver glycogen content in an obese postmenopausal rat model

The OVX group had a significantly lower level of liver glycogen (38.4 ± 4.6) than the control group (52.3 ± 6.4). That was further deteriorated by obesity induction in the OVX/HFD to a value 28.9 ± 3.9. In comparison to the other experimental groups, the irisin-treated OVX/HFD group showed a significant increase in liver glycogen to a value 67.5 ± 9.05 (Table 2).

### The effect of irisin on hepatic gluconeogenic enzymes in an obese postmenopausal rat model

In both the OVX and OVX/HFD groups, the main hepatic gluconeogenic enzyme activity, phosphoenolpyruvate carboxykinase (PEPCK) and glucose 6-phosphatase (G6Pase), was significantly increased as compared to the control group (Table 3). As obesity advances in the postmenopausal model, this assures a continuous rise in baseline hepatic glucose production and hyperglycemia.

**Table 3:** Effect of irisin on the metabolic enzyme activities of the liver, inguinal adipose, and muscle in an obese postmenopausal rat model

Enzymes activity	Group			
	Group I	Group II	Group III	Group IV
	Control	OVX	OVX/HFD	Irisin-treated OVX/HFD
Liver (PEPCK) (nmol/min/mg protein)	24.6 ± 3.4	38.4 ± 5.3^*^	48.7 ± 6.4^*#^	29.8 ± 5.4^§^
Liver (G6Pase)	33 ± 3.8	42 ± 4.3	56 ± 8.0^*#^	38 ± 5.2^§^
Inguinal fat (HSL) (nmol/min/mg protein)	24.80 ± 4.6	15.00 ± 2.5	18.40 ± 2.9^*^	40.50 ± 7.5^*§^
Inguinal fat (CPT) (nmol/min/mg mitochondrial protein)	2.45 ± 0.20	0.83 ± 0.13^*^	2.04 ± 0.22^#^	4.86 ± 0.89^*#§^
GM (CPT) (nmol/min/mg mitochondrial protein)	3.81 ± 0.64	2.49 ± 0.65^*^	3.24 ± 0.78	7.08 ± 1.4^*§^
Soleus (SDH) (nmol/min/mg protein)	2.750 ± 0.56	1.46 ± 0.22^*^	1.89 ± 0.23^*^	5.01 ± 1.12^*#§^
Soleus (CS) (nmol·min^−1^·mg protein^−1^)	310.8 ± 38.7	252.5 ± 28.9^*^	199.6 ± 20.3^*#^	558.8 ± 40.2^*#§^
Soleus COX (µmol/min/mg mitochondrial protein)	25.6 ± 4.9	17.4 ± 2.5^*^	19.6 ± 3.2^*^	56.7 ± 5.8^*#§^
GM (LDH) (nmol/min/mg protein)	912.3 ± 82.3	687.9 ± 45.3^*^	636.9 ± 34.4^*^	769.0 ± 34.7^#§^
GM Na^+^, K^+^-ATPase (nmol/mg protein/h)	122 ± 9.3	106.3 ± 8.9^*^	90.80 ± 8.5^*#^	113.7 ± 10.2^§^
Inguinal fat AMPK activity (fold change/control)	1	0.45 ± 0.02^*^	0.37 ± 0.01^*^	0.92 ± 0.18^#§^
GM AMPK activity (fold change/control)	1	0.53 ± 0.06^*^	0.44 ± 0.03^*^	0.89 ± 0.11^#§^

Values are expressed as mean ± SD of 10 rats in each group

^*^Significance vs. control group (*P* < 0.05)

^#^Significance vs. OVX group (*P* < 0.05)

^§^Significance vs. OVX/HFD group (*P* < 0.05)

*AMPK* AMP‐activated protein kinase, *CPT* carnitine palmitoyl transferase, *CS* citrate synthase, *COX* cytochrome c oxidase, *GM* gastrocnemius, *G6Pase* glucose 6-phosphatase, *HSL* hormone-sensitive lipase, *LDH* lactate dehydrogenase, *OVX* ovariectomized, *OVX/HFD* ovariectomized and high-fat diet fed group, *PEPCK* phosphoenolpyruvate carboxykinase, *Na*^+^*, K*^+^*-ATPase* sodium, potassium adenosine triphosphatase, *SDH* succinate dehydrogenase

In fact, irisin’s boosting effect on glycemic parameters (Table 1) was confirmed by a marked decline in hepatic PEPCK and G6Pase enzyme activity as compared to the OVX/HFD group (Table 3). This, along with the restoration of hepatic glycogen content as a result of irisin treatment (Table 2), confirmed the promoting irisin effect on the delicate balance between hepatic glucose production and peripheral storage to maintain the tightly regulated glucose homeostasis in the obese postmenopausal model under metabolic derangements.

### The effect of irisin on serum lipid profile in an obese postmenopausal rat model (Table 2)

Similarly, when compared to the OVX group, the lipid profile in the OVX/HFD group worsened, with further elevated blood levels of total cholesterol (TC), triglycerides (TG), and free fatty acids (FFA), while irisin treatment resulted in an observable decrease in serum lipid parameters.

### The effect of irisin on serum irisin level in an obese postmenopausal rat model

The induction of surgical menopause led to a significant reduction in irisin levels. The HFD/OVX rats consistently demonstrated more deterioration in irisin levels, which was recovered to a level comparable to the control group with continuous irisin therapy (Table 2).

### The effect of irisin on muscle substrate utilization preference (oxidative/glycolytic shift) in an obese postmenopausal rat model

The OVX group demonstrated a significant decrease in muscle carnitine palmitoyl transferase (CPT) enzyme activity, implying decreased long-chain FAs shuttling across the mitochondrial membrane and reduced FFA oxidation. The OVX/HFD group had higher CPT activity than the OVX group, while irisin therapy resulted in significantly higher CPT activity. Both the OVX and OVX/HFD groups had significantly decreased cytochrome c oxidase (COX), succinate dehydrogenase (SDH), and lactate dehydrogenase (LDH) activity compared to the control, indicating lower aerobic and anaerobic capabilities (Table 3).

**Fig. 3:**
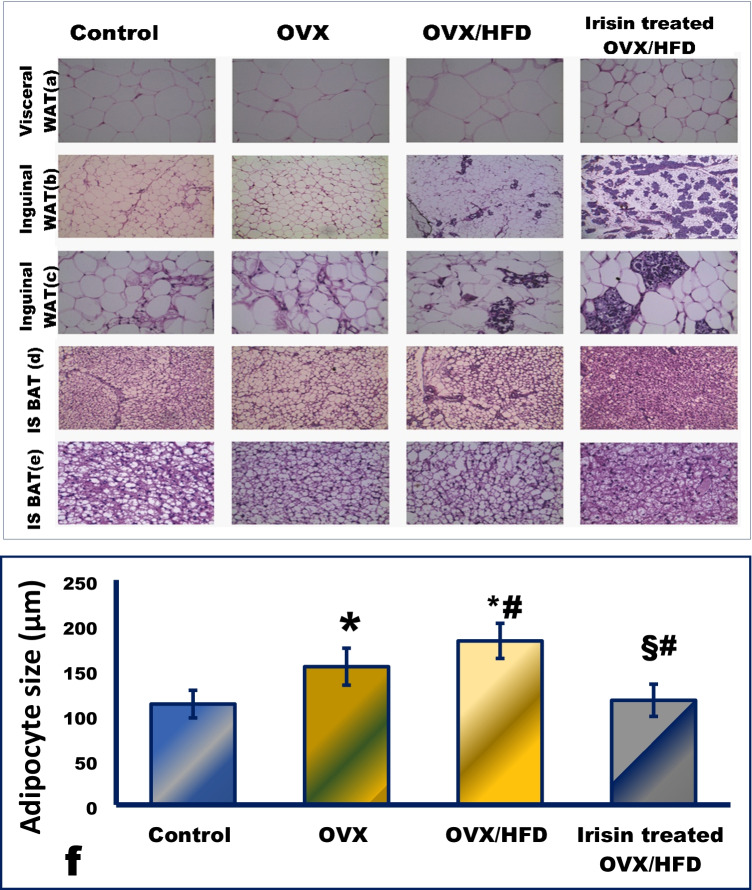
The effect of irisin on the morphological remodeling of different fat depots (**a**–**e**) and the size of adipocytes (f) in an obese postmenopausal rat model. Hematoxylin/eosin staining of visceral white adipose tissue (WAT, a), inguinal WAT (**b**, **c**), and the interscapular (IS) brown adipose tissue (BAT) (**d**, **e**). Quantifications of adipocyte size (**f**) from WAT. Magnification at ×100 (interscapular BAT, d), ×200 (inguinal WAT (b) and interscapular BAT (e)), and ×400 (visceral WAT (a) and inguinal WAT (c)). *Significance vs. control group (*P* < 0.05). #Significance vs. OVX group (*P* < 0.05). §Significance vs. OVX/HFD group (*P* < 0.05). The figure shows WAT from a control group that is typically composed of uniform cells with unilocular lipid droplets, eccentric nuclei, and few dispersed blood vessels, giving WAT its peculiar white-yellow appearance. Surgical induction of menopause (OVX group) is associated with increased adipocyte size, compared to the control group. The OVX/HFD group revealed a further increase in adipocyte size. Meanwhile, in the irisin-treated OVX/HFD group, there is a reduced size of adipocytes, reflecting decreased fat storage. a and b demonstrate a heterogeneous appearance in groups I and II, with both unilocular and multilocular adipocytes coexisting with interstitial tissue. OVX/HFD shows the appearance of nests of brownlike or multilocular adipocytes, whereas irisin-treated OVX/HFD shows a significantly increased proportion of these nests with a brown phenotype, indicating that irisin has a significant beiging effect. c and d show brown adipocytes (interscapular depots) from the control group reveal smaller polygonal cells with multilocular lipid droplets and central nuclei. The BAT from OVX and OVX/HFD groups reveals larger lipid droplets, indicating thermogenically quiescent BAT, while the irisin-treated OVX/HFD group obviously exhibits browning

**Fig. 4:**
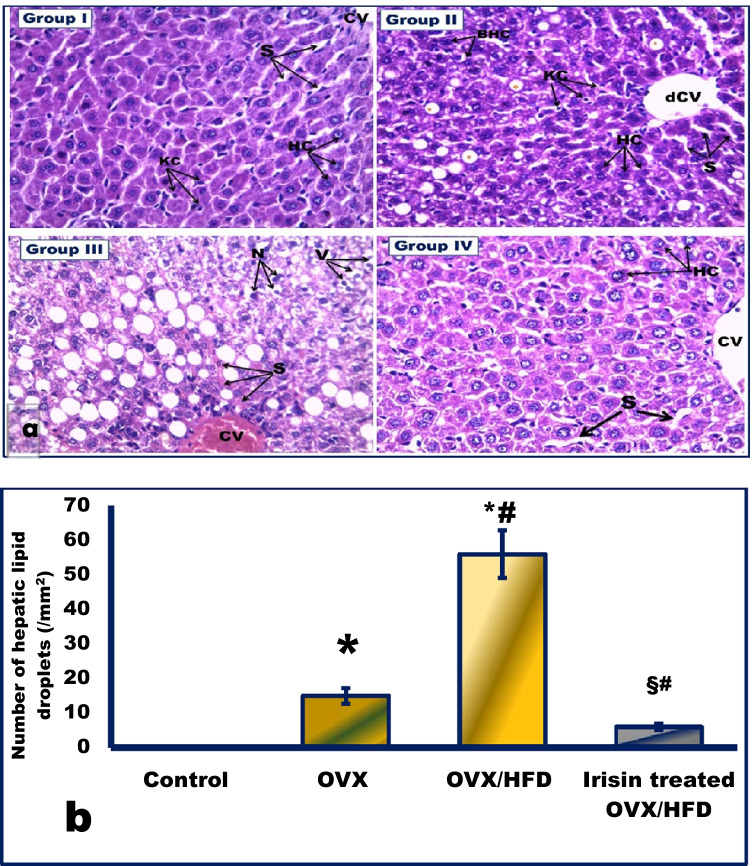
The effect of irisin on hepatic steatosis (**a**), and quantification of the number of hepatic lipid droplets per unit area (**b**) in an obese postmenopausal rat model. Representative photomicrographs of H&E stained liver sections in all experimental groups: the control group (group I) displays normal liver structure with polygonal hepatocytes (HC) with acidophilic cytoplasm and rounded vesicular nuclei radiating from the central vein (CV). Narrow radiating blood sinusoids (s) in between hepatic cords and their lining endothelium, Kupffer cells (KC), are noticed. The OVX group (group II) shows a dilated central vein (dCV). Binucleated hepatocytes (BHCs) are seen (arrow). The accumulation of lipid droplets (asterisks) in HC can be observed. The OVX/HFD group (group III) displays massive lipid steatosis with marked fatty degeneration of HCs with typical macrovesicular lipid droplets. Dilated, congested central vein (CV), and HCs with vacuolated cytoplasm (V) and darkly stained nuclei (N) that appear separated by congested blood (S) are also noticed. The irisin-treated OVX/HFD group (group IV) shows nearly complete clearance of steatohepatitis with restored hepatic histological architecture that appears similar to the control. Chords of normal The HCs radiate from the dCV and are separated by slightly dilated blood (S). Magnification × 400. *Significance vs. control group (*P* < 0.05). ^#^Significance vs. OVX group (*P* < 0.05). ^§^Significance vs. OVX/HFD group (*P* < 0.05)

**Fig. 5:**
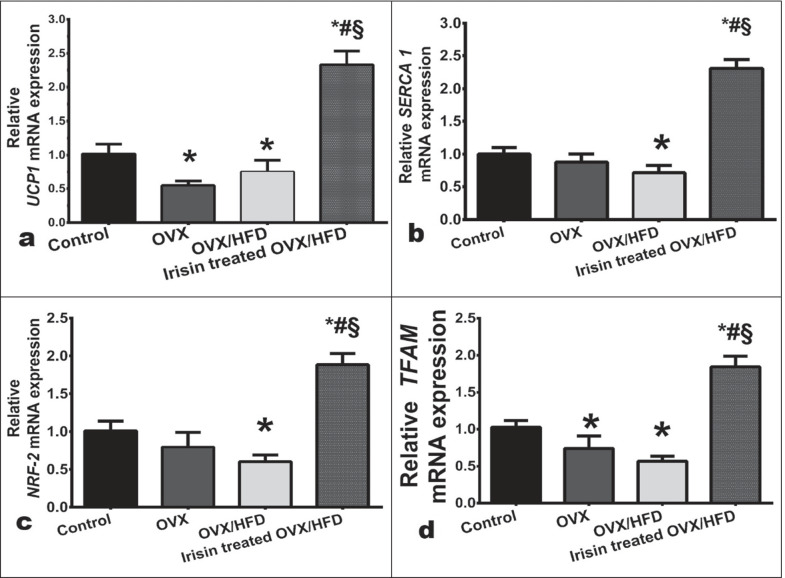
The effect of irisin on the relative gene expressions of **a**) adipose *UCP-1*, muscle **b**) *SERCA1*, **c**) *NRF-2*, and **d**) *TFAM* in an obese postmenopausal rat model. Values are expressed as the mean ± SD of 6 rats in each group. *Significance vs. control group (*P* < 0.05). ^#^Significance vs. OVX group (*P* < 0.05). ^§^Significance vs. OVX/HFD group (*P* < 0.05). UCP1 was measured from inguinal fat depot. Other gene expressions were assayed from the extensor digitorum longus. *NRF-2* nuclear respiratory factor erythroid 2‐related factor 2, *OVX* ovariectomized, *OVX/HFD* ovariectomized/high-fat diet fed group, *SERCA1* sarcoplasmic reticulum Ca^2+^-ATPas, *TFAM* mitochondrial transcription factor A, *UCP1* uncoupling protein

The irisin supplementation enhanced LDH levels and boosted SDH activity, indicating a shift toward higher oxidative potential and less reliance on glycolysis, particularly with increased mitochondrial content and COX activity (Figs. 3, 4, and 5).

### The effect of irisin on hormone-sensitive lipase (HSL) and CPT activity of inguinal fat in an obese postmenopausal rat model (Table 3)

CPT and HSL activities were measured to explore the possible effect of irisin on the rate-limiting enzyme in long-chain fatty acyl-CoA uptake and oxidation in mitochondria, as well as the lipolytic potential of fat depots.

In the OVX group, induction of menopause was linked with a substantial decrease in inguinal CPT activity (0.83 ± 0.13) compared to 2.45 ± 0.20 in the control group. When compared to the OVX group, obesity induction in the OVX/HFD group was linked with a higher CPT level. Irisin therapy boosted CPT activity to approximately 4.86 ± 0.89, which is substantially higher than the other experimental groups.

Similarly, HSL activity was lower in the OVX group than in the control group, while it was higher in the OVX/HFD group than in the OVX. When compared to the other experimental groups, HSL was elevated even more in the irisin-treated OVX/HFD group (Table 3).

### The effect of irisin on biomarkers and signal pathway of muscle mitochondrial biogenesis/content in an obese postmenopausal rat model

**Fig. 6:**
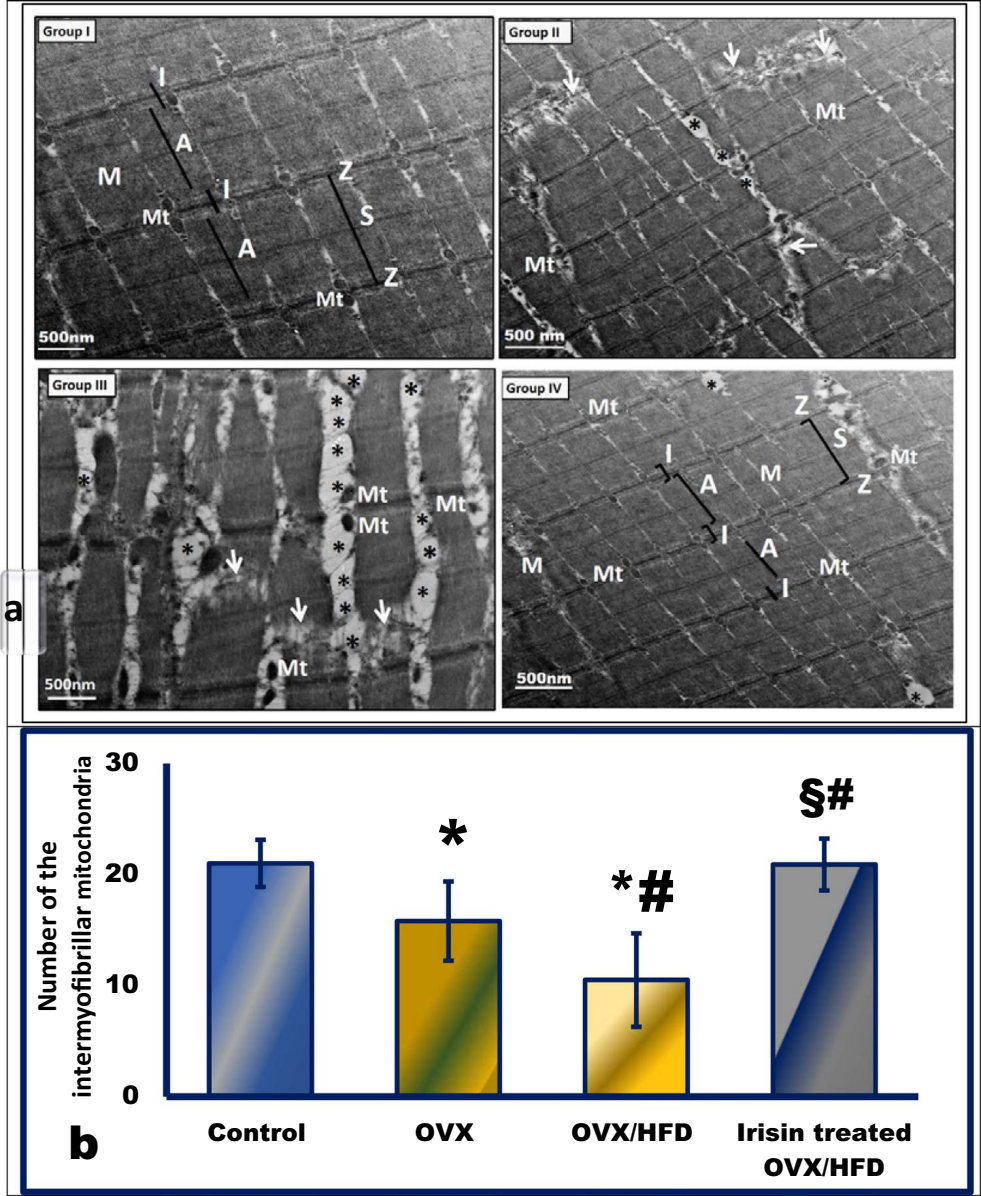
The effect of irisin on skeletal muscle ultrastructure (**a**) and quantification of the number of intermyofibrillar mitochondria (**b**) in an obese postmenopausal rat model. Representative electron micrographs of longitudinal ultrathin sections of the tibialis anterior (TA) muscle, from all experimental groups. TA from group I (control group) displays a normal myofibril banding pattern with alternating dark (A) and light (I) bands. The M and Z lines appear in the middle of the A and I bands, respectively. Sarcomeres (S) are extended between two successive Z lines. Mitochondria (Mt) appear in between myofibrils. TA from group II (OVX rats) shows the appearance of the lipid droplets (asterisks) as well as the reduced number of degenerated mitochondria (Mt), focal areas of myofibril degeneration (arrow) in aged skeletal muscle. Group III (OVX/HFD group) displays a higher density of large-sized lipid droplets (asterisks) adjacent to the distorted mitochondria (Mt). Distorted myofibrils’ banding with focal areas of degeneration (arrow). Group IV (irisin-treated OVX/HFD group) reveals greatly preserved muscle structure with normally shaped mitochondria (Mt) distributed in between the organized sarcomeres (S), while small dispersed lipid droplets (asterisks) can be noticed in between some disorganized myofibrils. Magnifications: the control and OVX/HFD groups (× 5000), while OVX and irisin-treated OVX/HFD groups (× 3000). *Significance vs. control group (*P* < 0.05). ^#^Significance vs. OVX group (*P* < 0.05). ^§^Significance vs. OVX/HFD group (*P* < 0.05)

Our findings suggested that surgically inducing menopause reduced indices of muscle mitochondrial content and density, and that obesity reduced them even further. Citrate synthase (CS) activities in the OVX (252.5 ± 28.9) and OVX/HFD (199.6 ± 20.3) groups were decreased compared to the control (310.8 ± 38.7). These results were confirmed by muscle ultrastructure (Fig. 6). When compared to the OVX/HFD group, irisin therapy increased skeletal muscles’ mitochondrial biogenesis and CS activity in postmenopausal obese rats (Table 3) (Fig. 6).

To explore the underlying signal transduction involved in the potential irisin effect, the expression levels of the transcriptional regulator of mitochondrial biogenesis, the peroxisome proliferator-activated receptor-gamma co-activator-1alpha (*PGC1α*) protein, as well as nuclear respiratory factor erythroid 2‐related factor 2 (*NRF-2*) and mitochondrial transcription factor A (*TFAM*) gene expressions were assayed. OVX/HFD rats resulted in downregulated expression levels of PGC1α protein (Fig. 8e), *NRF-2* (Fig. 5c), and *TFAM* (Fig. 5d) genes compared to the control, while irisin displayed marked upregulation in their expressions.

*The effect of irisin on muscle expression of total (t) and phosphorylated (p) mitogen-activated protein kinases (MAPK), and AMP-activated protein kinase (AMPK) activity, in an obese postmenopausal rat model.*

To further explore the underlying signaling network involved in the irisin effect on muscle, MAPK was assayed as the central metabolic mediator that promotes mitochondrial respiration and thermogenesis through orchestrating the expression level and activity of several transcription factors. Both the OVX and OVX/HFD rats displayed repression of t and p MAPK compared to the control group that were upregulated in the irisin-treated OVX/HFD group (Fig. 8c and d).

As a central sensor of energy homeostasis that coordinates several metabolic pathways and guides the delicate balances between energy demands and nutrient supply, the AMPK activity in muscle was assayed. As seen in Table 3, the OVX group had lower muscle AMPK activity than the control group. AMPK activity was much lower in the OVX/HFD group. Irisin therapy significantly increased AMPK activation to levels equivalent to controls.

### The effect of irisin on inguinal fat expression of t and p MAPK, and AMPK activity, in an obese postmenopausal rat model

**Fig. 7:**
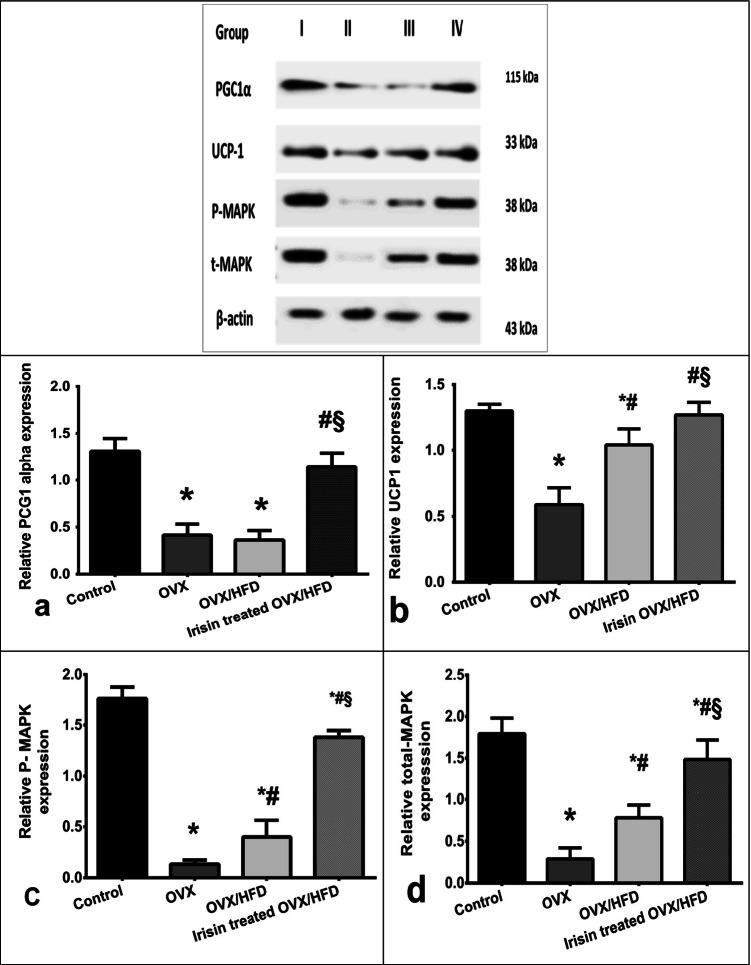
The effect of irisin on the inguinal adipose tissue protein expression in an obese postmenopausal rat model. Peroxisome proliferator-activated receptor-gamma co-activator-1alpha (PGC-1α, a), uncoupling protein 1 (UCP-1, b), phosphorylated mitogen-activated protein kinases (P-MAPKs, c) and total MAPKs (t-MAPK, d), in the control (group I), OVX (II), OVX/HFD (III), and the irisin-treated OVX/HFD (IV). Data are presented as means ± SD. *Significance vs. control group (*P* < 0.05). ^#^Significance vs. OVX group (*P* < 0.05). ^§^Significance vs. OVX/HFD group (*P* < 0.05). *OVX* ovariectomized, *OVX/HFD* ovariectomized/high-fat diet fed group

The expression levels of t and p MAPK were decreased in the OVX and OVX/HFD groups compared to the control group, as shown in Fig. 7c and d, while irisin therapy elevated MAPK levels considerably.

When compared to the control group, both the OVX and OVX/HFD groups had considerably lower AMPK activity, which was recovered by irisin therapy (Table 3).

### The effect of irisin on thermogenic parameters in adipose tissue in an obese postmenopausal rat model

The OVX group revealed significant downregulation of the protein level of mitochondrial UCP1 compared to the control. UCP1 was slightly upregulated with HFD challenge in group III, while it was significantly upregulated with irisin, in group IV, conferring a similar BAT phenotype (Fig. 6b).

Notably, OVX suppressed *UCP1* gene expression, but irisin was associated with upregulated *UCP1* level (Fig. 5a).

### The effect of irisin on thermogenic parameters in skeletal muscle in an obese postmenopausal rat model

**Fig. 8:**
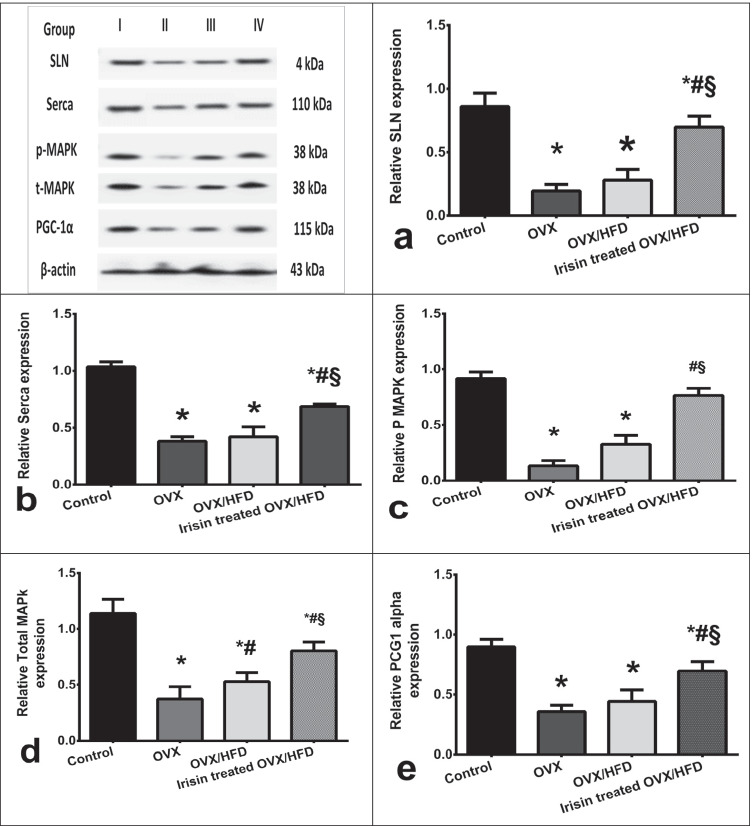
The effect of irisin on the muscle protein expression in an obese postmenopausal rat model. Sarcolipin (SLN, **a**), sarco/endoplasmic reticulum Ca^2+^-ATPase 1 (SERCA1, **b**), phosphorylated mitogen-activated protein kinases (P-MAPKs, **c**) and total MAPKs (t-MAPK, **d**) and peroxisome proliferator-activated receptor-gamma co-activator-1alpha (PGC-1α, **e**) in group I (control), II (OVX), III (OVX/HFD), and IV (irisin-treated OVX/HFD). Data is presented as a mean ± SD. *Significance vs. control group (*P* < 0.05). ^#^Significance vs. OVX group (*P* < 0.05). ^§^Significance vs. OVX/HFD group (*P* < 0.05). *OVX* ovariectomized, *OVX/HFD* ovariectomized/high-fat diet fed group

To explore the possibility of irisin as a tool for increasing EE in skeletal muscle, we investigated at its influence on SLN/SERCA-1 expression levels (Fig. 8a, b). Our data revealed a significant decrease in SLN/SERCA1 expression levels in the OVX group, which was worsened by HFD. Irisin increased the expression of SLN and SERCA1. Notably, the OVX and OVX/HFD groups had lower Na^+^/K^+^-ATPase activity than the control group. Furthermore, irisin increased skeletal muscle Na^+^/K^+^-ATPase activity (Table 3), resulting in increased energy consumption.

### The effect of irisin on muscle ultrastructure in an obese postmenopausal rat model (Fig. 6)

The OVX group showed areas of mitochondrial and focal myofibril degeneration that were aggravated in the OVX/HFD group. The ectopic intramuscular lipid infiltration was apparent in the OVX/HFD group compared to the OVX and the control groups, while irisin treatment resulted in a marked reduction in the lipid accumulation as portrayed in Fig. 6.

### The effect of irisin on hepatic structure in an obese postmenopausal rat model (Fig. 4)

In the OVX and OVX/HFD groups, the hepatic structure was distorted. In Fig. 4, hyperlipidemia in groups II and III was linked with significant lipid infiltration in the liver. A substantial rise in hepatic TG levels in the OVX and OVX/HFD groups verified this (Table 2). Irisin therapy resulted in a significant reduction in hepatic TG content, as shown in Fig. 4 and Table 2.

### The effect of irisin on morphology of different adipose depots in an obese postmenopausal rat model

Irisin demonstrated differential effects on adipose tissue depots. Irisin induced an apparent beiging in the inguinal WAT (Fig. 3a, b) and flaring of BAT activities (Fig. 3c, d). Even inguinal subcutaneous (WAT) displayed a remarkable degree of induced plasticity/remodeling with induced brown phenotype. Further details are in supplementary material.

### The effect of irisin on exhaustive swimming exercise in an obese postmenopausal rat model

The exhaustive swimming test was considerably impaired in the OVX and OVX/HFD groups, but irisin improved muscular performance in the swimming test in group IV (Table 2).

## Discussion

The current work highlights the full thermogenic irisin effect on the OVX obese rats with dissection of the muscle metabolic and functional profiles, besides its role in promoting browning in fat depots.

Previous studies have revealed age-dependent muscular atrophy, weakness, hyperphagia, reduced LBM, and EE with a net weight increase, as indicated in our data in the OVX groups [11, 12]. The observed decrease in HSL activity in OVX rats is consistent with the reduced adipose cell lipolysis and expansion of adipose tissue mass observed in our study.

Age-related obesity and metabolic dysfunction have been linked to reduced mitochondrial content and complex IV activity, which leads to decreased FA oxidation and, as a result, adipocyte hypertrophy, an effect that is predominantly driven by hypoxia-inducible factor-1-mediated complex IV repression [3]. The molecular axes that mediate age-dependent WAT enlargement may be linked centrally to hypothalamic downregulation of nicotinamide adenine dinucleotide-dependent deacetylase sirtuin 1 expression in agouti-related peptide neurons or over-activation of mammalian target of rapamycin signaling in pro-opiomelanocortin neurons in the arcuate nucleus [3, 13].

The OVX-associated metabolic dysfunction and altered anthropometric parameters were accompanied by lowered irisin levels, ensuring age-specific irisin reduction. The key determinants of circulating irisin are age and muscle mass. The statistically substantial negative correlation between irisin levels and muscular weakness and/or atrophy earlier corroborated this concept [11].

As an anabolic steroid, estradiol (E2) may enhance muscle mass through either upregulating irisin expression or directly driving irisin secretion. As a result, E2 withdrawal during menopause can merely moderate the effects of aging on irisin levels, particularly with decreased muscle mass, which contributes up to 72% of the total irisin pool [14]. The advanced age-related downregulation of skeletal muscle PGC-1 might account for, at least in part, the lower irisin levels in groups II and III. Irisin-treated OVX/HFD had higher levels of irisin, which might be linked to increased PGC-1, the primary inducer of irisin secretion.

Furthermore, irisin has been shown to upregulate PGC‐1α expression, which in turn tightly controls the *NRF-2* transcription and its downstream target (*TFAM*) and drives irisin transcription itself in skeletal muscle, suggestive of a positive feedback mechanism by which irisin might control its own expression [15].

Irisin levels have been found to be lower in type 2 diabetes mellitus (T2DM) patients [16], overweight/obese children with metabolic syndrome [1], and the HFD-induced obese mouse model [17]. High FNDC5/irisin levels, on the other hand, were related with a better metabolic profile and a lower risk of developing T2DM in obese middle-aged males [4].

The current results highlighted a potential irisin effect on feeding behavior, with slightly increased food intake that appears to be secondary to the associated increase in EE. Interestingly, prior evidence speculated that irisin might be an anorexigenic agent, through mechanisms that might involve appetite-regulating factors such as brain-derived neurotropic factor, amphetamine regulated transcript, orexins, and UCP2 [18].

Obesity induction, according to the current findings, exacerbated the OVX-induced deterioration in the metabolic profile. The observed hepatic glucose overproduction due to increased gluconeogenesis is a major contributor to the observed hyperglycemia in groups II and III. However, reduced glucose uptake by skeletal muscle could not be ruled out [19].

Group III hyperlipidemia is caused in part by impaired insulin-mediated suppression of lipolysis in adipose tissue, which leads to β-cell apoptosis and ectopic hepatic lipid deposition. This triggers hepatic inflammation and could play a key role in the development of IR [1, 20].

Interestingly, the current findings provide ample evidence of the promoting irisin effect on the metabolic milieu in OVX/HFD challenged rats. Indeed, under IR and streptozotocin/HFD-induced diabetes, both insulin and irisin have comparable downstream signal pathways in terms of enhancing lipid metabolism, boosting glycogenesis, and decreasing gluconeogenesis [1, 10, 17, 21]. This was corroborated by a significant decrease in the key enzymes involved in the hepatic gluconeogenic pathway.

Prior experimental evidence pointed to improved glucose homoeostasis under irisin treatment, by reducing gluconeogenesis via phosphatidylinositol 3-kinase (PI3K)/protein kinase B (Akt)/Forkhead box O-1-mediated PEPCK and G6Pase downregulation and boosting glycogenesis via PI3K/Akt/glycogen synthase (GS) kinase 3β-mediated (GS) activation [21].

The insulin sensitizing effect of irisin could be explained by enhanced GLUT4-mediated facilitated glucose uptake by skeletal muscles in an AMPK dependent manner, since AMPK activation has been shown to increase sarcolemmal and T-tubule GLUT4 translocation [18]. Irisin has also been identified as a pancreatic β-cell secretagogue and survival factor in lipotoxic conditions, potentially by decreasing endoplasmic reticulum stress [20, 22].

Irisin-mediated AMPK activation has been proposed as a way to mitigate the IR-associated metabolic deficits. AMPK activation has previously been shown to enhance glucose uptake and GLUT4 translocation in skeletal muscle [19]. AMPK activation lowers hepatic gluconeogenesis by directly suppressing G6Pase and PEPCK expression while enhancing CPT activity and FA oxidation [10, 19].

Irisin treatment significantly alleviated hepatic steatosis and dyslipidemia. It might be related to irisin’s ability to inhibit hepatic cholesterol and TG synthesis via AMPK-dependent inhibition of sterol regulatory element-binding transcription factor 2 and its downstream target genes [23].

Kim et al. [24] reported that increased circulating irisin levels were associated with better metabolic health indices in postmenopausal women on an endurance aerobic exercise program. The increased irisin level in the trained postmenopausal women was linked with lower waist circumference, TG, systolic blood pressure, and levels of HDL cholesterol [24].

Vliora et al. [25] recently revealed that irisin has a time-dependent role in the induction of mitochondrial NST, oxidative capability, lipolysis regulation, and EE in adipocytes. Irisins’ action is thought to be related to modification of multiple irisin downstream pathways, including PI3K-AKT, nuclear factor kappa B, and cAMP-response element binding protein [25].

Circulating irisin levels were negatively correlated with total cholesterol, LDL cholesterol, TG, and intrahepatic TG content in obese adults [22, 26], while they were positively correlated with HDL cholesterol [23].

The reduced molecular thermogenic parameters in inguinal fat depots in OVX and OVX/HFD groups were associated with downregulated P^38^ MAPK levels and reduced MAPK/AMPK activities. The isolated WAT/BAT from obese and insulin-resistant rodents and humans had lower AMPK activity, which was enhanced by obesity therapy [27, 28].

The promotion of the thermogenic phenotype of fat depots in the irisin-treated group might improve their capacity to effectively dissipate energy via mitochondrial (UCP-1)-mediated thermogenesis from Fas/glucose, limiting substrate availability for storage and potentially enhancing whole-body EE.

It has been demonstrated that HF diet dramatically decreased the expression of *PGC-1α*, *FNDC5*, and *UCP-1*, as well as palmitate oxidation and AMPK activity, in the inguinal fat depot [29]. Chronic AMPK activation, on the other hand, protects against HFD-induced obesity via UCP1 dependent and independent mechanisms [2].

Indeed, p^38^ MAPK has been identified as a key mediator in thermogenesis in adipose tissue and mitochondrial respiration in muscle through orchestrating the expression and activity of many transcription factors. P^38^ MAPK evokes upregulated *UCP1* expression through phosphorylation of the PGC-1α, and activating transcription factor 2, which interacts with PPAR and cAMP response elements that reside within the UCP1 gene promoter [30].

The evidence from animal and in vitro studies suggested that the induction of the brown fat-like phenotype by FNDC5/irisin was linked with a significantly better metabolic profile and raised EE [6]. Swick et al. [31] have observed a positive correlation between circulating irisin levels and 24-h EE in postmenopausal women. It has previously been demonstrated that knocking down or selective suppression of AMPK [10] and p^38^ MAPK [10] greatly reduced the irisin browning impact in cultured myocytes and diabetic mice [32]. As a result, irisin appears to drive browning via p^38^ MAPK/AMPk and *PGC-1α* axis, which guarantees not only the full UCP1 activation but also the maintenance of the brown-like phenotype of adipocytes.

Our finding of SLN downregulation in groups II and III matches in vivo evidence previously reported from SLN knockout mice [33] and obese individuals, which may be driven by the obesity-induced alteration in SR phospholipid milieu [34].

SERCA is the second largest energy-consuming protein in skeletal muscle. It has been identified as a major NST regulator. The altered kinetic properties and reduced activity of the SERCA have been implicated in the age-related decline in muscle strength. This might be attributable to selective nitration of the SERCA, most notably at Tyr^294^-Tyr^295^, which results in lower Ca^2+^ binding affinity or altered conformation of the SERCA nucleotide-binding domain, resulting in reduced force output and slowed contraction and relaxation [35]. Other evidence implicates the age-dependent oxidation of SERCA specific cysteine residues in the partial loss of SERCA1 functions in aging muscles [36].

Our findings are consistent with prior studies showing that stabilizing SERCA activity with elevated SLN and/or SERCA protein itself successfully reverses the muscle atrophy and weakness in aged models and significantly upregulates the oxidative metabolism [5, 36].

Away from the disruption of the metabolic profile in the skeletal muscle, the poor muscle performance, observed in OVX groups, might be explained by a decrease in anaerobic and aerobic enzyme activities as well as protein content [37]. Meanwhile, muscle remodeling and compositional changes associated with aging cannot be ruled out [36, 38].

The improved performance in the irisin-treated group might be attributed to increased SERCA activity that leads to increased cytosolic Ca^2+^. The higher cytosolic Ca^2+^ can maintain SR Ca^2+^ load, resulting in higher activation of myosin cross bridges and force production. Furthermore, Ca^2+^ acts as a metabolic signal to improve oxidative capacity in addition to activating the Ca^2+^ sensitive metabolic enzymes, which can boost ATP formation during high physiological demand such as exercise. Higher LDH may assure more ATP availability in group IV, as it catalyzes pyruvate conversion to lactate, thus promoting glycolysis and ATP production [5].

Supporting the notion that SLN is a key determinant of the BMR, SLN overexpressed mice maintained their normal metabolic profile and gained less weight in spite of increased caloric intake. SLN-knockout mice have reduced respiratory rates and are more prone to diet-induced obesity [33, 39]. Moreover, SLN overexpression displayed a higher endurance capacity and improved muscle performance by enhancing oxidative capacity [5, 39].

The results obtained from the AMPK mouse genetic models identified NRF-2 and PGC-1 as candidate downstream mediators of AMPK activation in mediating its influence on enhancing skeletal muscle mitochondrial density [19].

## Conclusion

While much progress has been achieved in decoding the physiological activities of irisin, we identified it for the first time as a possible thermogenic adipomyokine, by targeting the NST in the largest organs in the body, adipose tissue and skeletal muscle. Irisin enhanced EE, in the whole-body metabolic milieu. These findings put forward irisin as a new avenue to mimic or augment the effects of exercise in the treatment of postmenopausal obesity.

## Supplementary Information

Below is the link to the electronic supplementary material.

Supplementary file1 (DOCX 84 KB)

## Data Availability

R.A.E.G. will provide all data that support the current study’s findings upon request.
