# Correction: Irisin improves adiposity and exercise tolerance in a rat model of postmenopausal obesity through enhancing adipo-myocyte thermogenesis

**DOI:** 10.1007/s13105-023-00963-3

**Published:** 2023-04-28

**Authors:** Rehab E. Abo El Gheit, Reham L. Younis, Mervat H. El-Saka, Marwa N. Emam, Nema A. Soliman, Rehab M. El-Sayed, Yasser Mostafa Hafez, Norhan Ahmed AbuoHashish, Doaa A. Radwan, Howayda E. Khaled, Samar Kamel, Sawsan A. Zaitone, Ghada A. Badawi

**Affiliations:** 1 Faculty of Medicine, Department of Physiology, Tanta University, El Geesh Street, Tanta, Egypt; 2 Faculty of Medicine, Medical Biochemistry Department, Tanta University, Tanta, Egypt; 3 Faculty of Pharmacy, Department of Pharmacology & Toxicology, Sinai University, North Sinai, El-Arish, Egypt; 4 Faculty of Medicine, Internal Medicine Department, Tanta University, Tanta, Egypt; 5 Faculty of Medicine, Pharmacology Department, Tanta University, Tanta, Egypt; 6 Faculty of Medicine, Anatomy and Embryology Department, Tanta University, Tanta, Egypt; 7 Faculty of Sciences, Zoology Department, Suez University, Suez, Egypt; 8 Faculty of Veterinary Medicine, Physiology Department, Suez Canal University, Ismailia, Egypt; 9 Faculty of Pharmacy, Department of Pharmacology & Toxicology, Suez Canal University, Ismailia, 41522 Egypt; 10 Faculty of Pharmacy, Department of Pharmacology & Toxicology, University of Tabuk, Tabuk, 71451 Saudi Arabia

**Correction to: Journal of Physiology and Biochemistry (2022) 78:897–913**

**https://doi.org/10.1007/s13105-022-00915-3**

The original version of this article unfortunately contained an incorrect affiliation for Author Howayda E. Khaled “Zoology Department, Faculty of Science, Suez Canal University, Ismailia, Egypt” should be “Zoology Department, Faculty of Sciences, Suez University, Suez, Egypt.”

