# Supplementary material for: Irisin improves adiposity and exercise tolerance in a rat model of postmenopausal obesity through enhancing adipo-myocyte thermogenesis
Source: J Physiol Biochem. 2022 Aug 23;78(4):897–913. doi: 10.1007/s13105-022-00915-3 (PMC9684260; doi:10.1007/s13105-022-00915-3)
Supplement: Supplementary file 1 — Supplementary file1 (DOCX 84 KB) [file 13105_2022_915_MOESM1_ESM.docx]

Irisin improves adiposity and exercise tolerance in a rat model of postmenopausal obesity through enhancing adipo-myocyte thermogenesis

(Thermogenic irisin effect in postmenopausal obesity)

Rehab E. Abo El Gheit^1^, [Reham L Younis](https://pubmed.ncbi.nlm.nih.gov/?term=Younis+RL&cauthor_id=33833047)^1^, [Mervat H El-Saka](https://pubmed.ncbi.nlm.nih.gov/?term=El-Saka+MH&cauthor_id=32107872)^1^, Marwa N. Emam^1^, [Nema A. Soliman](https://onlinelibrary.wiley.com/action/doSearch?ContribAuthorStored=Soliman%2C+Nema+A)^2^ , Rehab M. El-Sayed^3^, Yasser Mostafa Hafez^4^, Norhan Ahmed AbuoHashish^5^, Doaa A Radwan^6^, Howayda E. khaled^7^, Samar Kamel^8^, Sawsan A. Zaitone^9,10^, Ghada A. Badawi^3^

^1^Department of Physiology, Faculty of Medicine, Tanta University, Tanta, Egypt.

^2^Medical Biochemistry Department, Faculty of Medicine, Tanta University, Egypt.

^3^Department of Pharmacology & Toxicology, Faculty of Pharmacy, Sinai University, El-Arish, North Sinai, Egypt.

^4^Internal Medicine Department, Faculty of Medicine, Tanta University, Tanta, Egypt.

^5^Pharmacology Department, Faculty of Medicine, Tanta University, Tanta, Egypt

^6^Anatomy and Embryology Department, Faculty of Medicine, Tanta University, Tanta, Egypt.

^7^Zoology Department, Faculty of Science, Suez Canal University, Ismailia, Egypt.

^8^Physiology Department, Faculty of Veterinary Medicine, Suez Canal University, Ismailia, Egypt.

^9^Department of Pharmacology & Toxicology, Faculty of Pharmacy, Suez Canal University, Ismailia 41522, Egypt

^10^Department of Pharmacology & Toxicology, Faculty of Pharmacy, University of Tabuk, Tabuk 71451, Saudi Arabia

Corresponding author:

Rehab El Sayed Abo El Gheit

Assistant professor of physiology

Address: Physiology Department, Tanta University, El Geesh Street, Tanta, Egypt

Tel: (00201029595331)

E-mail: [rehab.abouelghait@med.TanTa.edu.eg](mailto:rehab.abouelghait@med.TanTa.edu.eg)

[extrasystole.2003@gmail.com](mailto:extrasystole.2003@gmail.com)

**Research Design and Methods:**

**Exhaustive swimming exercise**

At the end of 8 weeks of irisin treatment protocol, all animals were fasted for 12 hours, then their exercise performance was evaluated in term of the swimming time, as described previously [9] .

The exercise performance was evaluated by forcing rats to swim against a load (5% of their body weight) fastened to their tail for 60 min. Each rat was evaluated in a columnar swimming pool (65 cm high, 20 cm diameter) with water 40 cm deep maintained at 27 ֯C. The endurance of each rat was measured as the swimming time, recorded from the beginning of swimming to exhaustion that was determined by observing loss of coordinated movements and failure to swim and return to the surface within 7 seconds. The swimming period was considered the time spent floating, struggling, and making necessary movements until exhaustion and possible drowning.

**Measurement of anthropometrical and obesity-related parameters**

At the end of the study, the length of each animal was measured, while the rats were anaesthetized, by a plastic non extensible measuring tape. The length of each rat was measured from the base of the lower incisors to the anus. Body mass index (BMI) for each rat, as an indicator of obesity, was calculated by the following formula: BMI (gm/cm^2^) = Final BW/length^2^. The cutoff value of obesity BMI is more than 0.68 gm/cm^2^ [21].

**Estimation of the biological value of the experimental diets:**

The biological value of the different experimental diets was assessed as follow; the animals were weighed at the first day of the experiment (initial weight), once weekly, and on the last day of experiment (final weight). Delta (BW) gain was calculated by subtracting the final BW of each rat from its initial BW [19].

BW gain percent (BWG %) =

Final BW – Initial BW (Delta BW) x 100.

Initial BW

**Food intake** in each cage was measured daily (5 days /week) during the light period starting at 8:00 AM prior to feeding of the experimental diets, in all groups for energy intake calculation. This value divided by 2 to obtain the mean consumption value for each rat. Fresh chow was provided daily and remaining amount from the previous day was collected, weighed and discarded. Food consumption was determined as the difference between the food supplied and the amount of food left in the grid [14].

**Metabolic energy** of standard ND and HFD were 3573 and 5404 Kcal/ Kg respectively. Calorie (energy) intake of consumed diet was estimated by multiplying the average consumed diet (in grams) by 3.573 and 5.404 respectively [14].

**Caloric efficiency** was expressed as gain in the BW divided by the total calorie intake from the start to the end of the experiment (Change in BW (g)/ gram of diet consumed) [15].

### Rectal temperature assay

Core body temperature was measured rectally with a thermistor (Micro-Therma 2 T/Thermo Works), inserted approximately 3 cm from the anus into the rectum, once weekly, during the light cycle.

**Animals’ dissection and calculation of organ and lean body masses (LBMs, fat-free)**

The body composition was measured by dissection technique, the gold standard for assessment of differential body composition, as illustrated previously [21]

Briefly, the rats were decapitated. An anterior longitudinal skin incision from neck to tail was performed. The entire skin was detached by scalpel consisting of fur and subcutaneous (WAT) from the carcass of each rat. Similarly the skin from the head was removed .The body and head skins were weighed separately. The classical inter-scapular (BAT) was carefully removed, cleaned from any visual (WAT). The thoracic and abdominal cavities were longitudinally incised and the internal organs were exposed. The heart, kidneys and liver were carefully removed and individually weighed. The lungs and the remaining abdominal organs were accounted for as viscera. Then all fat pad compartments such as inguinal (two superficial fat depots underneath the skin and anterior to the upper part of the hind limbs), retroperitoneal (distinct depot located around each kidney along the lumbar muscles), and the ovarian (fat surrounding uterus, ovaries and bladder) were individually removed and weighed. Their values were expressed in grams (g) prior to being processed for any further analysis [14]

The mass of the remaining carcass consisting of skeletal muscle and bones combined with the skinned head, liver, heart, and kidneys was used as LBM, the single best predictor of resting EE [21]

**Blood and tissues sampling and processing**:

**Blood sampling:** At the end of experiment, 24 hour after the exhaustive swimming exercise test, all rats were subjected to an overnight fast, to avoid the acute exercise effects on the measured parameters [21]. The rats were anesthetized with 30 mg/Kg^-1^ of 1% sodium barbiturate, I.P., followed by measurement of the anthropometric parameters. The rats were sacrificed by decapitation. Abdominal incision was immediately done and the blood samples were collected from the inferior vena cava, allowed to coagulate at room temperature and serum was separated by centrifuge at 3500 rpm for 15 minutes using a benchtop centrifuge (Centurion Scientific Limited). Clear sera were collected and stored at -80°C for subsequent biochemical.

**Tissue samples collection:** After careful dissection and weighting, the adipose tissue depots were instantly fixed in freshly prepared 4% paraformaldehyde (PFA) [wt/ vol] for 24 h, in 0.1 M phosphate buffer, pH 7.2, then proceeded for further histological analysis. BAT samples were collected from inter-scapular BAT depot. The inguinal WAT was equally divided into three portions. The first two portions were preceded to histological, biochemical analysis respectively. While the third portion of inguinal WAT together with the extensor digitorum longus (EDL) muscle samples were immediately placed on dry ice, homogenized (using a hand-held tissue homogenizer (Cole-Parmer LabGen 7), then divided into aliquots and stored at −80 °C and processed for western blot analysis.

The soleus muscle and entire gastrocnemius (GM) without separating red or white fibers were removed from right hind limbs together with the inguinal WAT samples were homogenized (using Precellys24Tissue Homogenizer, Bertin Technologies, France) in a solution of, 50 mM Tris–HCl, at pH 7.4 containing 0.01 mM phenylmethylsulfonyl fluoride and 1 mM EDTA. Homogenates were used for enzyme activities.

**Preparation of tissue homogenate**

(10–15 mg) of snap-frozen muscle and inguinal fat samples were homogenized in a fresh batch of homogenization buffer (10% glycerol, 20 mM sodium pyrophosphate, 1% NP-40, 20 mM b-glycerophosphate, 150 mM NaCl, 50 mM HEPES, 1 mM EDTA, 2 mM Na_3_VO_4_, 10 mM NaF, 2 mM phenylmethanesulfonyl fluoride, 1 mM EGTA, 10 µg/mL leupeptin, 10 µg/mL aprotinin and 3 mM benzamidine), in Precellys24Tissue Homogenizer (Bertin Technologies, France). Samples were rotated for one hour and then centrifuged using a cooling centrifuge (Model 3-30k, Sigma, USA) (16 500 g × 30 min) at 4 °C. Lysates were divided into aliquots and then stored at -80 °C for further analysis.

**Preparation of nuclear and membrane fractions**

Nuclear and membrane extracts from GM muscles were prepared using the Nuclear/Cytosol Fractionation Kit (BioVision, Inc., CA, USA, Cat# K266-100,) according to the manufacturer’s protocol, using a micro centrifuge (IKA mini G, India).

**Mitochondrial isolation [6, 18]**

Portions of GM muscles and inguinal fat were homogenized on ice, using Precellys24Tissue Homogenizer (Bertin Technologies, France), in mitochondrial isolation buffer (0.0001M EDTA-2Na, 0.01M Tris-HCl, 0.8% NaCl, 0.01M sucrose, pH 7.4). The homogenates were centrifuged using a cooling centrifuge (Model 3-30k, Sigma, USA) (1500 rpm × 5 min), at 4 °C. The resultant supernatants were then re-centrifuged, using a cooling centrifuge (Model 3-30k, Sigma, USA), (10.000 rpm × 15 min) to obtain the mitochondrial fraction.

**Biochemical analysis**

**Serum biochemical parameters:**

**Serum glucose and insulin levels** were measured using rat glucose ([Cat# MBS7233226](https://www.mybiosource.com/rat-elisa-kits/glucose/7233226)) (BTS 350 semiautomatic analyzer, Spain) and insulin (Cat # [MBS724709](https://www.mybiosource.com/ins-rat-elisa-kits/insulin/724709)) enzyme-linked immunosorbent assay (ELISA) Kits respectively, obtained from My BioSource, San Diego, USA. (Stat Fax®2100, Fisher Bioblock Scientific, France at 450 nm with correction wavelength set at 570 nm).

**Insulin resistance (IR) was measured by the homeostasis model assessment of insulin resistance (HOMA-IR),** a well-established marker for IR, that was calculated according to the following formula [6] .

HOMA‐IR = (fasting insulin level (µlU/mL) ˣ fasting glucose level (mg/dL)/405)

**Lipid profiles:** colorimetric assay using BTS 350 semiautomatic analyzer, Spain was done for measuring the levels of total cholesterol (TC) (Cat.# 10007640) and triglyceride (TG, in serum and liver), by enzymatic assay kits (Cat.#10010303, Cayman Chemical), as well as free fatty acids (FFAs) by the FFAs quantification kit (Cat.# ab65341, Abcam, MA, USA) [6].

**Serum irisin** was assayed using commercial irisin ELISA kit (EK-067–16; Phoenix Pharmaceuticals, Inc.; Burlingame, CA) [8]. All assays were carried out following the manufacturer’s instruction, using (Stat Fax®2100, Fisher Bioblock Scientific, France at 450 nm with correction wavelength set at 570 nm).

**Tissue biochemical analysis**

**Hepatic phosphoenolpyruvate carboxykinase (PEPCK) and glucose-6-phosphatase (G6Pase) activity assay [13]:** Hepatic PEPCK and G6Pase activities were measured immediately within 3 h of tissue removal, using an NADH-coupled system to quantitate the phosphoenolpyruvate conversion into oxaloacetate and subsequently to malate through using malic dehydrogenase. The conversion was then monitored by a reduction in NADH absorbance at wave length 340 nm. Activity was expressed as nmol per min per mg protein (BTS 350 semiautomatic analyzer, Spain).

**Glycogen assay**: Glycogen content in GM muscle and liver samples was measured colorimetrically, using (BTS 350 semiautomatic analyzer, Spain) [17]. Briefly, liver and muscle tissues were weighed and then completely dissolved by their addition to (30% KOH (300 μL), Na_2_SO_4_). The homogenized solution was mixed with ethanol (360 μL). The homogenate was placed, for 30 min, on ice, then centrifuged (15 min, 5000×g, 4 °C). The supernatant was removed. The glycogen-containing precipitates were dissolved in distilled water. Sulfuric acid and Phenol were added. The mixture was left for 15 min to react. The absorbance was assayed at 490 nm. The results were expressed as mg/g tissue.

**Succinate dehydrogenase (SDH) activity assay**: SDH as an index of the oxidative metabolic capacity, was determined from soleus muscle[16], using (BTS 350 semiautomatic analyzer, Spain). Briefly, the soleus muscle was homogenized through a glass tissue homogenizer (pH 7.4., 5 volumes of ice-cold 0.3 M phosphate buffer). Sodium succinate was then added to approach (17 mM) as a final concentration. The final concentrations of the different components of the reaction mixture were as follows: sodium cyanide (1 mM), sodium succinate (17 *μ*M), calcium chloride (0.4 mM), and aluminum chloride (0.4 mM). The reduction of cytochrome *c* was then assayed observing the increased extinction at (550 nm). SDH activity was expressed as nmol/min/mg protein.

**Citrate synthase (CS) activity assay*:* (**CS) as a marker of mitochondrial content / density, was assayed by spectrophotometric detection of reduced thionitrobenzoic acid, from soleus muscle, using (BTS 350 semiautomatic analyzer, Spain). Briefly, about (900 μl) of reaction mixture [50 μM EDTA, 0.5 mM oxaloacetate, 0.1 mM 5,5-dithiobis-(2-nitrobenzoic) acid (DTNB), 0.31 mM acetyl CoA, 0.1M Tris/HCL, and 5 mM triethanolamine hydrochloride] at (pH 8.1) was mixed. Then (100 μl) of isolated mitochondria was subsequently added and CS activity was measured at wavelength of (405 nm). CS activity was expressed as nmoles/min/mg protein.

**Cytochrome c oxidase (Complex IV (COX)** activity **[12]**: COX activity was measured in the isolated mitochondria from soleus muscle, by monitoring the maximal oxidation rate of the fully reduced cytochrome c as described previously [10], using (BTS 350 semiautomatic analyzer, Spain). Briefly, rates of enzyme reaction were assayed at room temperature (25 ˚C) through assay the change in absorbance of the reduced to the oxidized cytochrome c, at wave length (550 nm). COX activity was expressed as µmol / min/ mg protein.

**Lactate dehydrogenase (LDH) activity.** LDH as a measure of the glycolytic capacity [11], was measured, from GM muscle. LDH activity was assayed by utilizing the chromogenic indicator, as a tetrazolium salt, reflecting the dehydrogenase activity [7], using (BTS 350 semiautomatic analyzer, Spain). Briefly, LDH was assayed in potassium phosphate [50 mM at pH 7.2, 2.1 mM pyruvate, 100 μM NADH, 1 mM EDTA] and 10 μl of supernatant. The NADH, substrates, and pyruvate were immediately added before assaying of LDH activity, and the reaction was then started. The results were expressed as nmol/min/mg protein.

**Total Carnitine palmitoyl transferase (CPT, both CPTI, and CPTII) activity:** the rate-limiting enzyme in long-chain fatty acyl- CoA uptake and oxidation in mitochondria, was evaluated in the freshly isolated mitochondria from GM and inguinal fat [11], using (BTS 350 semiautomatic analyzer, Spain). Briefly, the reaction mixtures were composed of [1.5 mM EDTA, 0.05% Triton X-100, 60 mM Tris–HCl, at (pH 8.0), 1.67 mM carnitine, and 0.25 mM DTNB]. About 100 µL of supernatant together with the substrates carnitine and DTNB were immediately added before measuring the enzyme activities. The reaction was commenced through the addition of [0.025 mM] palmitoylCoA. The results were expressed as (nmol) /min/mg protein.

**Hormone-sensitive lipase (HSL) activity assay**: [20]

Inguinal fat depots were dissected out, minced, and homogenized in 10 volumes of medium containing 1 mM EDTA, 0.25 M sucrose, 1 µg. ml^-1^ pepstatin A, 20 µg. ml^-1^ leupeptin and 1 mM dithiothreitol (pH 7.0). Then homogenates were centrifuged at 105,000 g for 45 min at 4֯C. (Using a Beckman TL-TB-023B ultracentrifuge with an MLS-50 Rotor (Beckman CoulterTM, CA, USA). The fat cake was then removed. After centrifugation, pellets containing crude membranes of inguinal fats were used. The fat-free infranatant was recovered for analysis of maximal activity of enzyme, using 1(3)-mono-[3H] oleoyl-2-O-oleylglycerol as substrate. All samples were incubated at 37°C, for 30 minutes. HSL activity was expressed as (nmol) / min/mg protein.

**Muscle Na^+^/K^+^ -ATPase activity assay:** [2] Colorimetric quantification of the Na^+^, K^+^-ATPase activity in membrane fractions, isolated from GM, was performed using (BTS 350 semiautomatic analyzer, Spain). Na^+^, K^+^-ATPase activity was assayed by the ATP hydrolysis and the Pi-molybdate complex production. Na^+^/K^+^ ATPase activity was expressed as nmol/mg protein/h.

**Muscle and adipose AMPK activity assay:** GM and inguinal fat depots samples were individually incubated at 30°C, for 30 min, in a pre-coated plate with a substrate peptide that corresponded to mouse insulin receptor substrate/1 that is efficiently phosphorylated through AMPK. AMPK activities were assayed by semi-quantitative immunoassay AMPK Kinase Assay kit (Cat# CY-1182, CycLex Co., Ltd, Nagano, Japan), according to the manufactures protocol. AMPK activities were quantified by assaying the IRS/1 phosphorylation at Ser^789^, using phosphor/Ser^789^ IRS/1 monoclonal anti-mouse antibody and peroxidase linked IgG. Substrate conversion was quantitated by detecting the absorbance at wave length 450 nm, (Stat Fax®2100, Fisher Bioblock Scientific, France) as a fold change relative to the control

**Measurement of protein contents**: Total protein contents in the tissue and mitochondrial samples were assayed by BCA assay (Pierce, Rockford, IL USA). (BTS 350 semiautomatic analyzer, Spain).

**Tissue morphometric and muscle ultra-structural analyses**

**Light microscopy**: after fixation, the fat depots (brown, beige, and white), and liver were embedded in paraffin. To assess the morphological changes, sections were processed for hematoxylin and eosin (H&E) staining using a binocular digital microscope (SCO Tech GmbH, Germany [21]. The average size of the adipocytes was calculated using the digital imaging software program “ImageJ” (ImageJ, National Institutes of Health, Bethesda, USA, RRID: SCR_003070). For each group, the mean adipocyte size was calculated in 20 adipocytes from ten randomly selected fields. The number of lipid droplets per unit area in the liver was calculated in ten randomly selected fields from slides of each group. (˟400 magnifications).

**Electron microscopy (EM) ultra-structural of skeletal muscle [1]**

Tibialis anterior (TA) muscle samples were fixed in 5% glutaraldehyde overnight, at 4 ֯C, rinsed in 0.1 M phosphate buffered saline, pH 7.2. Ultrathin muscle sections were processed for (EM) examination by, a JEOL transmission electron microscope at 80 kV, at Electron Microscopy Unit, Faculty of Medicine, Tanta University. The average number of the intermyofibrillar mitochondria was counted in each group at 10 randomly selected fields at 3000 magnifications

**Western blot analysis of signaling proteins in muscle and adipose tissues [5]:**

Muscle homogenates were prepared from the whole frozen EDL muscle that was diluted (19:1 (vol/wt) in an ice-cold buffer (20 mM Tris, 2 mM EDTA, 135 mM NaCl, 2 mM DTT, , 2 mM sodium pyrophosphate, 25 mM β-glycerophosphate, 1 mM sodium orthovanadate, 10% glycerol, 1% Triton X-100, 10 mM NaF, 10 mg/mL aprotinin, 1 mM PMSF, and10 mg/mL leupeptin, pH 7.5) and then centrifuged at 12000 g for 30 min at 4 °C.

For beige inguinal WAT, about 100 mg was homogenized in lysis buffer (50 mM Tris–HCl, 5 mM EDTA, 150 mM NaCl, at pH 7.4), without Triton X-100. Homogenates were then centrifuged at 3000 g for 15 min (4°C). After removal of the fat cake from the top of the tube, Triton X-100 was added at a final 1% concentration, then incubated for 30 min at 4°C, and centrifuged at 15,000 g × 15 min, at 4°C.

Total protein content in supernatant was quantified using a BCA kit (Pierce, Rockford, IL USA).  50 μg protein from tissue lysates were loaded in each well of a 12% sulfate-polyacrylamide gel electrophoresis. After electrophoresis, proteins were transferred to polyvinylidene fluoride membranes (Carl Roth, Karlsruhe, Germany). Membranes were blotted with the indicated primary antibodies overnight at 4°C, as follow; polyclonal rabbit anti-SLN antibody (Cat# ABT13, EMD Millipore, Burlington, MA, USA), mouse IgG monoclonal anti-SERCA1 ATPase (VE121G9, cat# ab2819, Abcam); anti- Phosphorylated p^38^ MAPK (Thr^180^/Tyr^182^) (cat# 4511, Cell Signaling Technology), total p^38^ α/β MAPK (cat#sc-7972), anti- PGC-1α (4A8) (cat#sc-517380) from (Santa Cruz Biotechnology, Santa Cruz, CA), rabbit polyclonal anti UCP-1 (cat# ab23841, Abcam) and the control monoclonal anti–β-actin (Cat#sc-517582, Santa Cruz Biotechnology, Santa Cruz, CA). The primary antibodies were used according to manufacturer’s recommendations. After washing with Tween in Tris-buffered saline, the membranes were incubated for one hour with Horseradish peroxidase goat anti rabbit IgG antibodies (Abcam, cat# [ab6721)](https://www.abcam.com/goat-rabbit-igg-hl-hrp-ab6721.html), at room temperature. Immune complexes were detected by the enhanced chemiluminescence method (TMA-100, Lumigen technology, Bioquote Limited, UK). Protein bands were quantified by was scored using a digital imaging software program “ImageJ” (ImageJ, National Institutes of Health, Bethesda, USA, RRID: SCR_003070), normalized to β-actin band density [4].

**Quantitative estimation of relative gene expressions by real-time reverse transcription PCR (RT-PCR) [3]**

Qiagen RNeasy Total RNA isolation kit (Qiagen, Hiden, Germany) was used to extract total RNA from frozen EDL muscle and inguinal adipose depot according to the manufacturer’s protocols. SuperScript ® III First‐StrandSynthesis System for RT‐PCR kit (Life Technologies) was used after the first step to form the first strand. Power SYBR Green PCR Master Mix (Life Technologies) was used to carry out PCR reactions. Target genes mRNA expression was assayed relative to the housekeeping gene, [Glyceraldehyde 3-phosphate dehydrogenase](https://en.wikipedia.org/wiki/Glyceraldehyde_3-phosphate_dehydrogenase) (GAPDH). Primers were designed by Primer3 software (http://bioinfo.ut.ee/primer3/) as follows: *nuclear factor erythroid 2–related factor 2 (Nrf2)* forward (F): (5′-TAGCAGAGCCCAGTGGCGGT-3′) and reverse (R): (5′-TGCTCTGGGGATGCTCGGCT-3′) (GenBank Accession No. [NM_031789.2](https://www.ncbi.nlm.nih.gov/nuccore/NM_031789.2)), ***SERCA1*** F: 5′-GACTGAGTTTGGGGAACAGCT-3′ and R:
5′-GAGGTGGTGATGACAGCAGG-3′ (GenBank Accession No. [NM_058213.1](https://www.ncbi.nlm.nih.gov/nuccore/NM_058213.1)), *mitochondrial transcription factor A (TFAM****)*** F: 5′-ATCAAGACTGTGCGTGCATC-3′ and R:
5′-AGAACTTCACAAACCCGCAC-3′ (GenBank Accession No. [NM_031326.1](https://www.ncbi.nlm.nih.gov/nuccore/NM_031326.1)), ***UCP1*** F: 5′- ACTGCCACACCTCCAGTCATT-3′ and R:
5′- CTTTGCCTCACTCAGGATTGG-3′ (GenBank Accession No. [NM_012682.2](https://www.ncbi.nlm.nih.gov/nuccore/NM_012682.2)) and GAPDH F: 5′‐GGTGAAGTTCGGAGTCAACGGA‐3′ and R:5′‐GAGGGATCTCGCTCCTGGAAGA‐3′ (GenBank Accession No. NM_017008). Relative gene expression was automatically calculated using the comparative threshold (Ct) method for the values of the target and the reference genes using the 2^-ΔΔCT^ formula by Rotor‐Gene Q 6plex and its specific software (Qiagen,Valencia, CA, USA).

Results

**Effect of irisin on morphology of different adipose depots in obese postmenopausal rat model**

Fig. (3) reflects the heterogeneity in the morphology of the different adipose depots and remodeling capacity to irisin treatment under HFD in OVX rats**.**

(Fig. 3) shows that WAT, that is typically composed of uniform cells with unilocular lipid droplets, eccentric nuclei together with few dispersed blood vessels, giving WAT its peculiar white/yellow appearance. Surgical induction of menopause was associated with increased adipocytes size, compared to the control group, that was further increased in OVX/HFD group. Meanwhile, irisin treatment reduced size of adipocytes reflecting decreased fat storage.

(Fig. 3a and b) reveals heterogeneous appearance in group (I, and II), displaying both uni/multilocular adipocytes together with interstitial tissue. Indeed, the HF challenge of OVX rats triggered the appearance of nests of brown-like / multi-locular adipocytes, while irisin treatment markedly increased the proportion of these nests with a brown phenotype ensuring prominent beiging effect of irisin.

In (Fig. 3c and d), the brown adipocytes from the interscapular depots reveal smaller polygonal cells with multi‐locular lipid droplets, and central nuclei. BAT from OVX group revealed larger lipid droplets, indicating thermogenically quiescent BAT. While irisin treatment obviously enhance the browning in HFD challenged OVX rats reflecting increased EE.

References;

1. Abo El Gheit RE, Atef MM, Badawi GA, Elwan WM, Alshenawy HA, and Emam MN. Role of serine protease inhibitor, ulinastatin, in rat model of hepatic encephalopathy: aquaporin 4 molecular targeting and therapeutic implication. *Journal of physiology and biochemistry* 2020.

2. Abo El Gheit RE, Atef MM, El Deeb OS, Badawi GA, Alshenawy HA, Elwan WM, Arakeep HM, and Emam MN. Unique Novel Role of Adropin in a Gastric Ulcer in a Rotenone-Induced Rat Model of Parkinson's Disease. *ACS chemical neuroscience* 11: 3077-3088, 2020.

3. Abo El Gheit RE, Soliman NA, Badawi GA, Madi NM, El-Saka MH, Badr SM, and Emam MN. Retinoprotective effect of agmatine in streptozotocin-induced diabetic rat model: avenues for vascular and neuronal protection : Agmatine in diabetic retinopathy. *Journal of physiology and biochemistry* 2021.

4. Abo El Gheit RE, Soliman NA, Nagla SA, El-Sayed RM, Badawi GA, Emam MN, Abdel Ghafar MT, Ibrahim MAA, Elswaidy NRM, Radwan DA, Alshenawy HA, Khaled HE, Kamel S, El-Saka MH, Madi NM, and Younis RL. Melatonin epigenetic potential on testicular functions and fertility profile in varicocele rat model is mediated by Silent information regulator1. *British journal of pharmacology* 2022.

5. Alomar SY, Gheit REAE, Enan ET, El-Bayoumi KS, Shoaeir MZ, Elkazaz AY, Al Thagfan SS, Zaitone SA, and El-Sayed RM. Novel Mechanism for Memantine in Attenuating Diabetic Neuropathic Pain in Mice via Downregulating the Spinal HMGB1/TRL4/NF-kB Inflammatory Axis. *Pharmaceuticals* 14: 307, 2021.

6. Atef MM, Abd-Ellatif RN, Emam MN, Amer AI, and Hafez YM. Therapeutic potential of sodium selenite in letrozole induced polycystic ovary syndrome rat model: Targeting mitochondrial approach (selenium in PCOS). *Archives of biochemistry and biophysics* 671: 245-254, 2019.

7. Babson AL, and Babson SR. Kinetic colorimetric measurement of serum lactate dehydrogenase activity. *Clinical chemistry* 19: 766-769, 1973.

8. Boström P, Wu J, Jedrychowski MP, Korde A, Ye L, Lo JC, Rasbach KA, Boström EA, Choi JH, Long JZ, Kajimura S, Zingaretti MC, Vind BF, Tu H, Cinti S, Højlund K, Gygi SP, and Spiegelman BM. A PGC1-α-dependent myokine that drives brown-fat-like development of white fat and thermogenesis. *Nature* 481: 463-468, 2012.

9. Chen WC, Huang WC, Chiu CC, Chang YK, and Huang CC. Whey protein improves exercise performance and biochemical profiles in trained mice. *Medicine and science in sports and exercise* 46: 1517-1524, 2014.

10. Fajardo VA, McMeekin L, Saint C, and LeBlanc PJ. Cardiolipin linoleic acid content and mitochondrial cytochrome c oxidase activity are associated in rat skeletal muscle. *Chemistry and physics of lipids* 187: 50-55, 2015.

11. Kaczor JJ, Ziolkowski W, Antosiewicz J, Hac S, Tarnopolsky MA, and Popinigis J. The effect of aging on anaerobic and aerobic enzyme activities in human skeletal muscle. *The journals of gerontology Series A, Biological sciences and medical sciences* 61: 339-344, 2006.

12. Li QOY, Soro-Arnaiz I, and Aragonés J. Age-dependent obesity and mitochondrial dysfunction. *Adipocyte* 6: 161-166, 2017.

13. Liu TY, Shi CX, Gao R, Sun HJ, Xiong XQ, Ding L, Chen Q, Li YH, Wang JJ, Kang YM, and Zhu GQ. Irisin inhibits hepatic gluconeogenesis and increases glycogen synthesis via the PI3K/Akt pathway in type 2 diabetic mice and hepatocytes. *Clinical science (London, England : 1979)* 129: 839-850, 2015.

14. Ludgero-Correia A, Jr., Aguila MB, Mandarim-de-Lacerda CA, and Faria TS. Effects of high-fat diet on plasma lipids, adiposity, and inflammatory markers in ovariectomized C57BL/6 mice. *Nutrition (Burbank, Los Angeles County, Calif)* 28: 316-323, 2012.

15. Maurya SK, Bal NC, Sopariwala DH, Pant M, Rowland LA, Shaikh SA, and Periasamy M. Sarcolipin Is a Key Determinant of the Basal Metabolic Rate, and Its Overexpression Enhances Energy Expenditure and Resistance against Diet-induced Obesity. *The Journal of biological chemistry* 290: 10840-10849, 2015.

16. Nishizaka T, Nagatomo F, Fujino H, Nomura T, Sano T, Higuchi K, Takeda I, and Ishihara A. Hyperbaric oxygen exposure reduces age-related decrease in oxidative capacity of the tibialis anterior muscle in mice. *Enzyme research* 2010: 824763, 2010.

17. Prestes J, Leite RD, Pereira GB, Shiguemoto GE, Bernardes CF, Asano RY, Sales MM, Bartholomeu Neto J, and Perez SE. Resistance training and glycogen content in ovariectomized rats. *International journal of sports medicine* 33: 550-554, 2012.

18. Qaisar R, Bhaskaran S, Ranjit R, Sataranatarajan K, Premkumar P, Huseman K, and Van Remmen H. Restoration of SERCA ATPase prevents oxidative stress-related muscle atrophy and weakness. *Redox biology* 20: 68-74, 2019.

19. Rising R, and Lifshitz F. Energy expenditures & physical activity in rats with chronic suboptimal nutrition. *Nutrition & metabolism* 3: 11, 2006.

20. Shih MF, and Taberner PV. Dose-dependent effects of chronic ethanol on mouse adipose tissue lipase activity and cyclic AMP accumulation. *British journal of pharmacology* 120: 721-727, 1997.

21. Wu MV, Bikopoulos G, Hung S, and Ceddia RB. Thermogenic capacity is antagonistically regulated in classical brown and white subcutaneous fat depots by high fat diet and endurance training in rats: impact on whole-body energy expenditure. *The Journal of biological chemistry* 289: 34129-34140, 2014.

| Table S1 Nutrient composition and ingredients of (grams per kilogram) of the experimental diets | | |
| --- | --- | --- |
| Contents(g) | ND | HFD |
| Cornstarch | 620.7 | 250.7 |
| Casein (≥85% protein) ) | 140.0 | 190.0 |
| Sucrose | 100.0 | 100.0 |
| Soybean oil | 40.0 | 40.0 |
| Fiber | 50.0 | 50.0 |
| Lard | ------- | 320.0 |
| AIN- 93 Mineral mix | 35.0 | 35.0 |
| AIN- 93 Vitamin mix | 10.0 | 10.0 |
| Choline | 2.5 | 2.5 |
| Antioxidant | 0.008 | 0.008 |
| L-Cystin | 1.8 | 1.8 |
| Energy intake/gm of diet | 3.573 | 5.404 |
